# Biochemical and Cellular Characterization of the Function of Fluorophosphonate-Binding Hydrolase H (FphH) in *Staphylococcus aureus* Support a Role in Bacterial Stress Response

**DOI:** 10.1021/acsinfecdis.3c00246

**Published:** 2023-10-12

**Authors:** Matthias Fellner, Annabel Walsh, Stephen Dela Ahator, Nadia Aftab, Ben Sutherland, Eng W. Tan, Alexander T. Bakker, Nathaniel I. Martin, Mario van der Stelt, Christian S. Lentz

**Affiliations:** † Biochemistry Department, School of Biomedical Sciences, University of Otago, Dunedin 9054, New Zealand; ‡ Research Group for Host-Microbe Interactions, Department of Medical Biology and Centre for New Antibacterial Strategies (CANS) UiT, The Arctic University of Norway, 9037 Tromsø, Norway; § Department of Chemistry, Division of Sciences, University of Otago, Dunedin 9054, New Zealand; ∥ Department of Molecular Physiology, Leiden Institute of Chemistry, Leiden University, 2333 CC Leiden, The Netherlands; ⊥ Biological Chemistry Group, Institute of Biology Leiden, Leiden University, 2333 BE Leiden, The Netherlands

**Keywords:** *Staphylococcus aureus*, lipases, esterase, serine hydrolases, biofilm

## Abstract

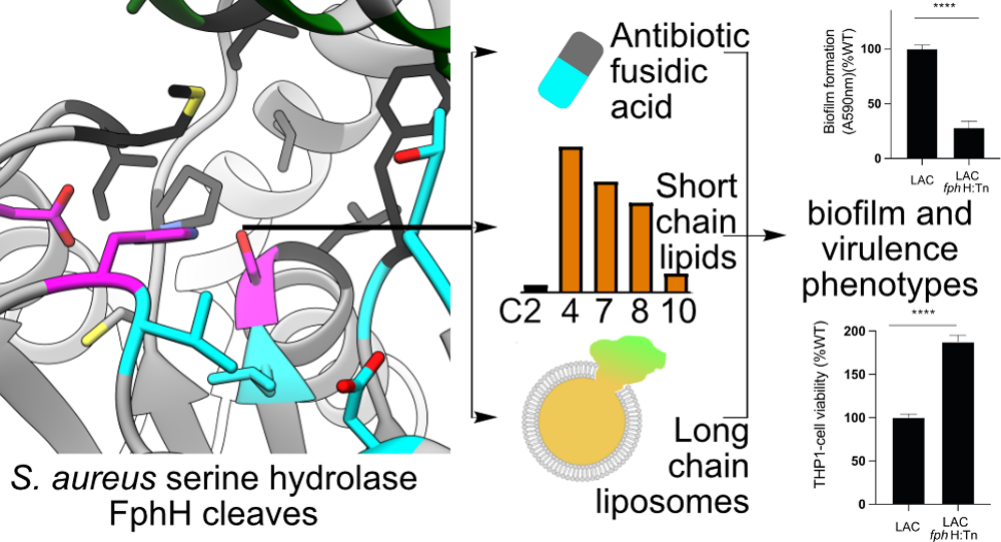

The development of new treatment options for bacterial infections requires access to new targets for antibiotics and antivirulence strategies. Chemoproteomic approaches are powerful tools for profiling and identifying novel druggable target candidates, but their functions often remain uncharacterized. Previously, we used activity-based protein profiling in the opportunistic pathogen *Staphylococcus aureus* to identify active serine hydrolases termed fluorophosphonate-binding hydrolases (Fph). Here, we provide the first characterization of *S. aureus* FphH, a conserved, putative carboxylesterase (referred to as *yva*K in *Bacillus subtilis*) at the molecular and cellular level. First, phenotypic characterization of *fph*H-deficient transposon mutants revealed phenotypes during growth under nutrient deprivation, biofilm formation, and intracellular survival. Biochemical and structural investigations revealed that FphH acts as an esterase and lipase based on a fold well suited to act on a small to long hydrophobic unbranched lipid group within its substrate and can be inhibited by active site-targeting oxadiazoles. Prompted by a previous observation that *fph*H expression was upregulated in response to fusidic acid, we found that FphH can deacetylate this ribosome-targeting antibiotic, but the lack of FphH function did not infer major changes in antibiotic susceptibility. In conclusion, our results indicate a functional role of this hydrolase in *S. aureus* stress responses, and hypothetical functions connecting FphH with components of the ribosome rescue system that are conserved in the same gene cluster across *Bacillales* are discussed. Our atomic characterization of FphH will facilitate the development of specific FphH inhibitors and probes to elucidate its physiological role and validity as a drug target.

*Staphylococcus aureus* presents a significant challenge to public health globally as the bacterial pathogen associated with the highest number of deaths in individuals older than 15 years and the second highest number of deaths associated with antimicrobial resistance.^1,2^ In particular, methicillin-resistant *S. aureus* (MRSA) was found to be responsible for more than 100,000 deaths in 2019.^1^ In a recent cell-based chemical proteomics study aimed at profiling key enzymes in *S. aureus*, we identified 12 enzymatically active serine hydrolases, with several predicted to be carboxylesterases.^3^ Follow-up studies^4−6^ demonstrated that the active site serine can be targeted by covalently binding small molecules. These findings make these enzymes promising new targets for inhibitor and probe development toward new drugs and diagnostics.^7^ Based on the fluorophosphonate probe that was initially used to discover these serine hydrolases, they are annotated as Fluorophosphonate-binding hydrolases (or Fph enzymes for short).^3^

In this study, we focus on the uncharacterized putative carboxylesterase FphH that belongs to the protein family IPR012354 Esterase/lipase in InterPro.^8^ In a recent bioinformatic analysis, we found that all staphylococcal species contain a FphH homologue.^9^ The Uniprot^10^ database lists 54 entries of 90% or higher sequence similarity to the *fphH* (Q2G025, SAOUHSC_00802) entry for *S. aureus* reference strain NCTC 8325/PS 47 (Table S1), all from other Staphylococci strains (mostly *S*. *aureus*), with one likely mis-annotated *Escherichia coli* entry. Across the commonly studied *S. aureus* strains NCTC 8325, COL, USA300 and Newman the sequence of *fphH* is 100% identical. In most of the 54 entries the gene is annotated as *est*, *est*2 or *yva*K, if no strain-specific gene locus is used. The gene is located in the gene cluster *secG-yva*K*(fph*H*)-rnr-smpB-ssr*A (SAUSA300_0761 – SAUSA300_0764) that has been characterized mostly in *Bacillus subtilis*, where the *fph*H gene has been annotated as *yva*K.^11^ In the following, we will refer to the *S. aureus* homologue as *fph*H and the *B. subtilis* homologue as *yva*K. The gene cluster is largely restricted to the order *Bacillales*, including *S. aureus* and *B*. *subtilis* with the shorter form *secG-yvaK-rnr-smpB* occurring within two taxa of the order *Lactobacillales, Lactobacillus plantarum* and *Enterococcus faecalis*.^12^ In *B. subtilis*, expression of *yva*K and the *rnr* gene which encodes Ribonuclease R is under the control of the general stress response regulator σ_B_ and is induced upon exposure to ethanol, salt, cold, or heat.^13−15^ In *E. coli*, *rnr* contributes to the cold shock response,^16^ whereas in *B. subtilis* null mutations in neither *rnr* nor *yva*K (*fph*H) affected temperature-dependent growth.^11^*S. aureus* Ribonuclease R is associated with RNA processing, decay, and ribosome quality control, but unlike the *E. coli* counterpart, it appears to not be regulated by general stressors and acetylation.^17^ SmpB acts with the Transfer mRNA (tmRNA) *ssr*A in the *trans-*translation system that rescues stalled ribosomes.^11^ In *B. subtilis*, *smpB* was required for growth at high temperature, while both *smpB* and *ssrA* were required for growth at low temperature.^11^ It has been suggested that the conserved gene order predicts that these dissimilar gene products together affect a common cellular process such as adaptation to cold, in an unknown way.^11^

Multiple *in vitro* studies have characterized homologues of this enzyme from various *Bacillus* or *Listeria* strains which share ∼50–55% sequence identity with FphH.^18−27^ In general, these esterases have been described as lipases/carboxylesterases with an affinity for shorter lipid chains, but the exact physiological function of *fph*H/*yva*K remains unknown. In our original chemoproteomic study, we identified FphH activity when growing *S. aureus* strains ATCC35556 and Newman under biofilm-promoting conditions on TSAMg Agar.^3^ Subsequently, we found that FphH activity is also found in *S. aureus* during exponential phase and is targeted by a novel oxadiazolone (referred to as “compound 3”) believed to inhibit FphH via a covalent mechanism.^28^ A previous study performed in strain SH1000 demonstrated that expression of *fph*H (described as carboxyl esterase *est*, SACOL0845) was over 10-fold up-regulated in response to the antibiotic fusidic acid.^29^ Fusidic acid is a protein biosynthesis inhibitor that blocks amino acid transfer from aminoacyl-tRNAs from the ribosome.^30^ In which way FphH may contribute to the functional response against fusidic acid is unclear.

In this study, we aimed to characterize the function of *S. aureus* FphH at both the molecular and cellular level. We phenotypically characterized *fph*H-deficient transposon mutant strains in a variety of *in vitro* growth and virulence-related assays, indicating diverse areas of potential physiological relevance. We determined the atomic structure of FphH and performed substrate profiling and inhibition studies with the recombinant enzyme, demonstrating its function as a carboxylesterase that can be inhibited by oxadiazolones. We found that FphH can deacetylate and thus inactivate fusidic acid, but transposon-inactivation of the *fph*H gene did not affect the susceptibility to fusidic acid in cells. Our results provide further evidence for the role of this enzyme in stress responses that may be linked to other components of the *secG-fphH/yvaK-rnr-smpB* gene cluster.

## Results

### Functional Characterization of FphH-Deficient Transposon Mutants

**Figure 1:**
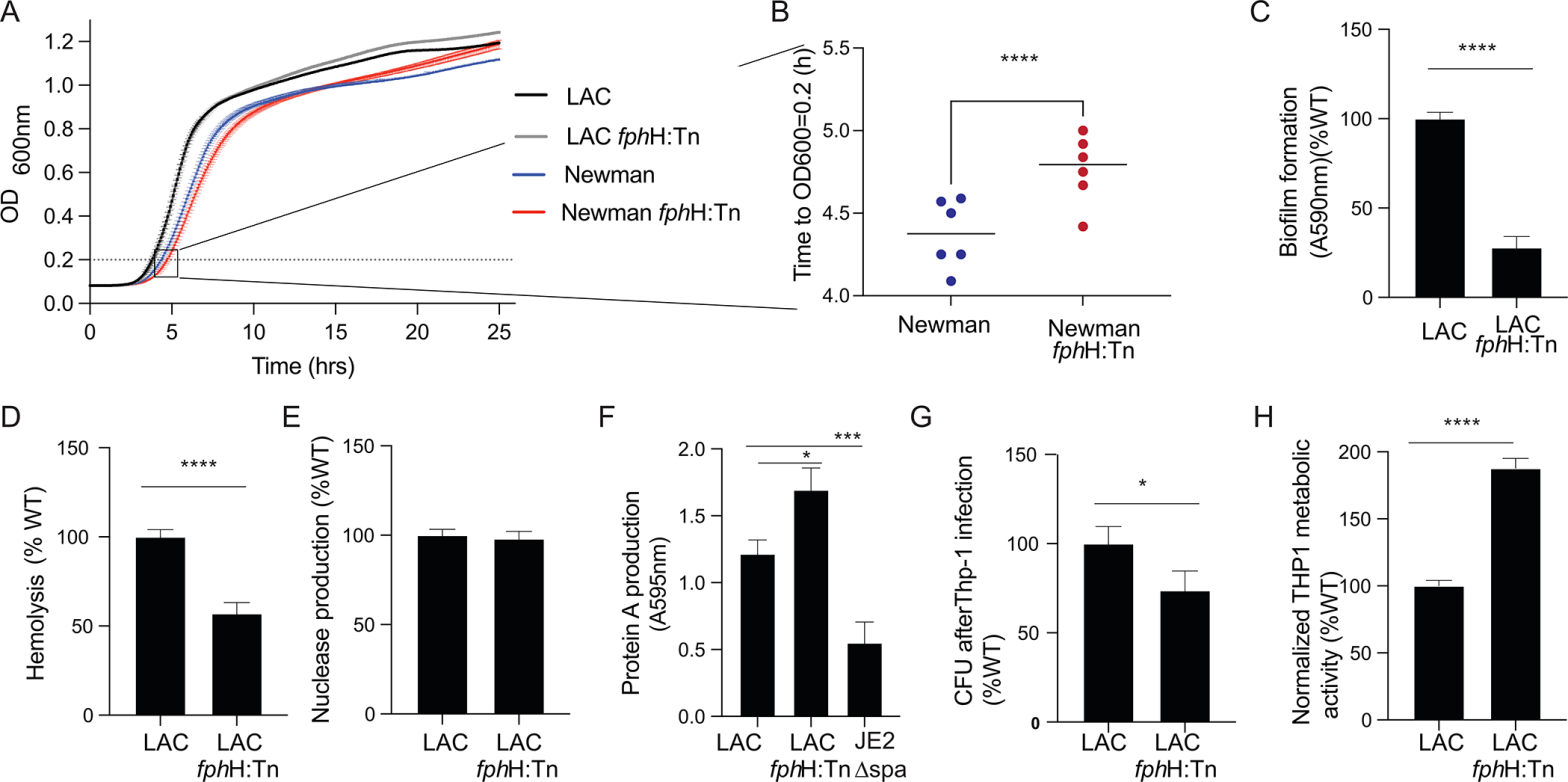
Phenotypic characterization of *S. aureus fph*H:Tn strains. A) The growth curve of the wild-type (WT) and *fph*H transposon mutant (*fph*H:Tn) strains based on strain Newman and USA300 (LAC), cultured in TSB in microplates. The graph shows means ± SEM of data from *n* = 6 biological replicates derived from two independent experiments. B) Increased lag of the Newman *fph*H:Tn growth curve compared to the WT curve (as shown in A) illustrated by the time to reach an OD600 = 0.2 (*n* = 6 independent biological replicates from 2 experiments). C) Relative biofilm formation as determined by a crystal violet assay. D) Hemolysis of red blood cells and E) nuclease production in the *fphH* mutant, as assessed in agar diffusion assays, are compared to those in the LAC WT. In C–E, the *fph*H:Tn values were normalized to the WT at 100%, with error bars representing the standard deviation (SD) based on *n* = 5 biological replicates. F) Protein A production is depicted for the WT, *fph*H:Tn, and protein A-deficient strain (Δspa). The graph displays the mean ± SD from *n* = 3 independent experiments. G) The relative colony forming units of the LAC WT and *fphH*:Tn after 45 min of infection of the human macrophage-derived cell line THP-1 (MOI = 10). Extracellular bacteria had been killed by gentamicin treatment, and intracellular bacteria were recovered after THP1 cell lysis and plated for CFU determination. H) The relative THP-1 cell viability following 1 h infection by the LAC WT and *fphH*:Tn (MOI = 10) as measured by the MTT assay. The values in G and H for the *fphH*:Tn were normalized to the WT at 100% with error bars indicating the SD of *n* = 3 biological replicates. In B–H, significance was tested by an unpaired, two-tailed Student’s *t* test.

To investigate the potential physiological role of FphH in *S. aureus*, we first tested transposon mutant strains derived from the Nebraska Transposon Mutant Library^31^ in growth assays. We observed that the *fph*H:Tn in Newman, but not USA300 (LAC) background, showed reduced growth in late exponential/early stationary phase (Figure 1A), whereas after 15 h the mutant cultures in both Newman and LAC backgrounds reached higher densities. In addition, the Newman *fph*H:Tn mutant showed an increased lag that was quantified as a statistically significant delay in reaching a threshold OD600 = 0.2 (Figure 1B). This increased lag may point to a slower adaptation to the new growth environment or indicate a deficiency in the previous stationary phase overnight culture that was used for inoculation. These growth phenotypes that manifest mainly under nutrient-limited conditions are in line with a previously observed compensatory upregulation of FphE^4^ (a homologous serine hydrolase of unknown function) in Newman *fph*H:Tn and suggest that FphH may play an important role in maintaining cellular activity/viability under stressful, nutrient-deprived conditions.

Next, we sought to functionally investigate the *fph*H mutant in a panel of *in vitro* virulence assays. Here, we focused on the Methicillin-resistant strain USA300 (LAC) as a prototype for the clinically relevant community associated-MRSA USA300 lineage.^32,33^ We found that the mutant strain showed reduced levels of biofilm formation (27.6 ± 6.1%) relative to that of the wild-type (WT; Figure 1C). We also tested for other common virulence traits that are ascribed to the production of specific virulence factors such as hemolysis (due to hemolysins) and nuclease activity (secreted nuclease) and levels of protein A production. The mutant strain showed decreased hemolysis areas (57.0 ± 6.1% of WT) (Figure 1D), no differences in the level of secreted nuclease activity (Figure 1E) and an increase in protein A production (139.0 ± 13%) (Figure 1F). Finally, we tested if the *fph*H:Tn mutant strain showed a fitness defect in the intracellular survival in macrophages. We observed that the *fph*H:Tn mutant showed a minor reduction in intracellular survival in the THP1 macrophage cell line (73.0 ± 11%) compared to WT (Figure 1G). However, THP1 cells cocultured/infected with *fph*H:Tn cells showed significantly higher levels of metabolic activity (188.0 ± 7%) as determined by MTT assay compared to those exposed to USA300 (LAC) WT cells (Figure 1H). Our results suggest that FphH may play a role in survival under stress conditions, in biofilms, and regulation of virulence traits. It remains to be determined whether the observed phenotypes are directly or indirectly attributable to the loss of FphH function or if they may be caused by secondary mutations. To account for potential polar effects due to transposon insertion, we have evaluated a JE2-based mutant with a transposon insertion in the *rnr* gene (downstream of *fph*H) on biofilm formation, hemolysis, THP-1 cell infection, and growth. Despite their shared operon, the *rnr* mutants exhibited a significant influence solely on biofilm formation, (Figure S1) suggesting that, in general, the phenotypes observed for the *fph*H:Tn strain are unlikely to be caused by polar effects. Having established potential areas of relevance at the cellular level, we proceeded with investigations of the function of FphH at the molecular level.

### Overall Characteristics and Structure of FphH

**Figure 2:**
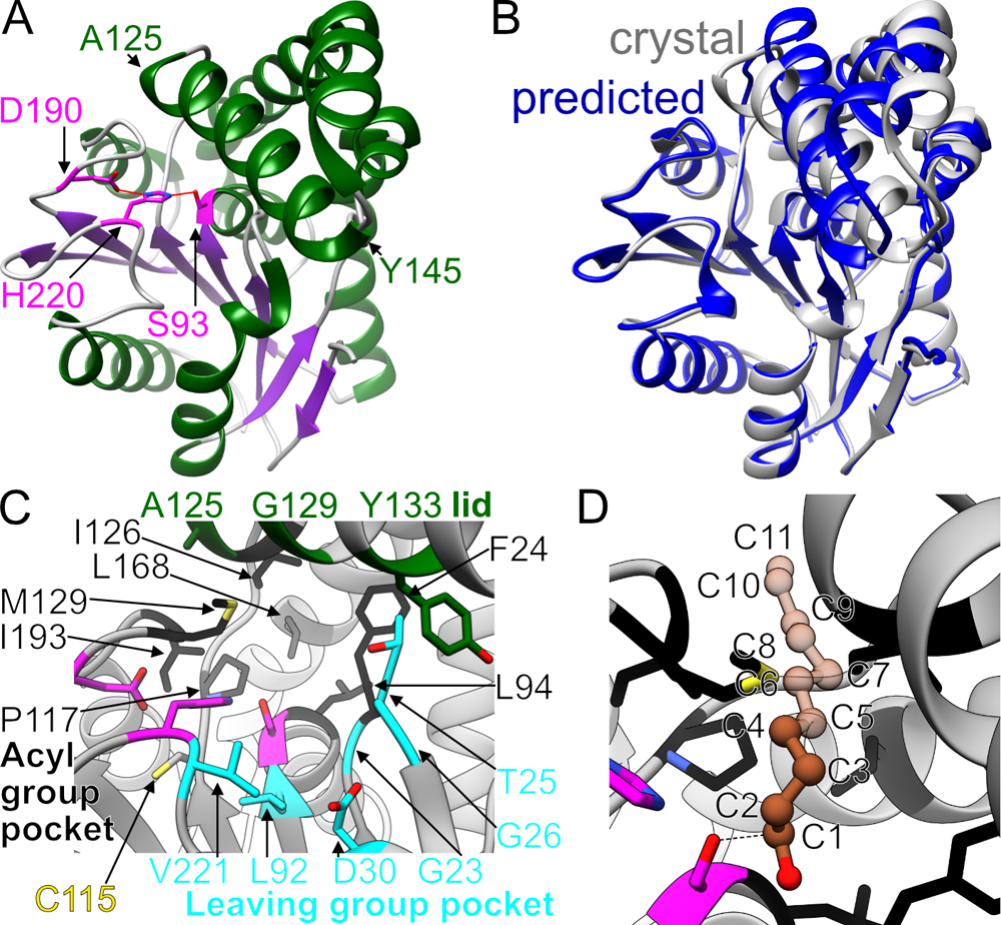
FphH structure. A) Ribbon representation of the FphH monomer (PDB ID 8FTP) showing the location of the active site triad. β-Strands are colored in green, α-helices in purple, and the active site Ser-His-Asp triad in magenta. Start and end of the active site lid helix, spanning residues 125 through 145, is indicated. B) Ribbon comparison of FphH crystal structure in gray and predicted Alphafold model in blue. C) Active site environment. Triad in magenta, acyl group pocket in black, leaving group pocket in cyan, modifiable cysteine in yellow, and potential interacting lid residues in green. D) Modeling of an unbranched lipid chain into the acyl group pocket based on C7-bound crystal structure of FphF (PDB ID 6WCX). Cα-alignment of FphH and FphF resulted in the placement of the C7 chain shown in the figure; C8–C11 atoms were added manually by continuing the lipid chain without additional minimization or refinement. C1–C4 in dark brown indicating well-defined pocket contacts compared to C5–C11 atoms with fewer and fewer contacts.

To gain insight into the molecular function of FphH, we first determined the atomic protein structure. We cloned the full length *fph*H sequence (SAUSA300_ 0763) using USA300 genomic DNA as a template into a pET28a vector for recombinant expression of the full-length 28 kD enzyme in *E. coli*, examined behavior in solution, and determined crystallization conditions. FphH crystallized in several different conditions (Table S1) spanning at least three different crystal forms. These crystal forms were very similar and always showed the same monomeric structure (Figure 2A), with oligomer analysis in PISA^34^ also suggesting no oligomer formation in solution. In solution, based on gel-filtration experiments (Figure S2), FphH elutes slightly later than a 44 kD protein standard but much earlier than would be expected for 28 kD. This still indicates that FphH appears to be a monomer in solution that interacts differently with the gel-filtration resin (cross-linked agarose and dextran) compared to most proteins. In addition, FphH behaves unlike molecular weight markers on SDS-PAGE, with the band appearing at approximately 35 kD (Figure S3), matching observations of the FphH SDS-PAGE band from *S. aureus* cell lysates.^3^ Interestingly, liquid chromatography (LC)-coupled electrospray high resolution mass spectrometry was unable to obtain a clean signal of the intact FphH protein, indicating many differentially modified forms of the same protein, like oxidations. Lower resolution MALDI-TOF intact protein measurement confirmed the ∼28 kD size (Figure S4A), also ruling out post-translational modification that would account for the size difference observed via gel-filtration or SDS-PAGE.

FphH crystals diffracted to higher resolution in the presence of calcium atoms with final models showing Calcium binding on the surface of FphH, especially at crystal contact sites (Figure S5). The data set resulting from the highest diffracting crystal at a resolution of 1.37 Å was deposited under PDB ID 8FTP. FphH is a member of the α/β hydrolase superfamily^35^ illustrated by the well conserved core structure of seven β-strands connected by several helices (Figure 2A). We used a model generated with Alphafold^36^ for molecular replacement of our initial structure and this model as well as our predicted FphH structural model discussed in our previous work^5^ closely match the overall fold of the experimentally determined crystal structure. However, there are notable differences between the predicted and experimental model with an RMSD of 1.3 Å across all Cα atoms of FphH. In particular, the helices connecting β4 and β5 (residues 115–181) fold significantly differently (Figure 2B). The B-factors are also the highest in the structure for these helices, indicating that this region is the most flexible (Figure S6).

### Active Site of FphH

The FphH serine hydrolase catalytic triad consists of Ser93, His220, and Asp190 (Figure 2A) in the usual H-bonding arrangement for this fold to enable the serine residue to cleave substrates that bind to the surrounding active site pockets. Ser93 is located within the conserved GXSXG motif.^37^ In contrast to some transmembrane prediction tools used to annotate FphH when it was first discribed,^3^ our structure findings show 85–105 is not a continuous putative transmembrane helix. This region contains the active site Ser93 residue, in a fold matching the α/β hydrolase superfamily,^35^ making cytosolic the most likely location for FphH. We previously called the substrate binding pockets in both sides of the active site serine for FphF as the acyl and leaving group binding pockets.^5^

FphH also contains a well-defined acyl binding pocket that is very hydrophobic, consisting of residues Phe24, Leu94, Pro117, Ile126, Leu168, Met192 and Ile193 (Figure 2C). This pocket is partially opened to the surface and suggests that FphH may also be able to act as a carboxylesterase similar to FphF (Figure 2D).^5^ The FphF pocket is optimally suited for a C7 fatty acid, with longer chains being blocked by side chains.^5^ Cα-alignment of FphF-C7-bound and FphH creates an FphH bound model that suggests that FphH is able to accommodate a C4 unbranched lipid chain bound in a similar way. In contrast to FphF, FphH is able to accommodate a longer chain in the narrow hydrophobic extension beyond the core acyl binding pocket (Figure 2D). Up to 11 carbon chain atoms would still have clear hydrophobic interactions with FphH residue side chains, with a longer lipid chain still being plausible as the end of the extension is surface exposed. The leaving group pocket is not as well-defined, showing a large surface exposed area that is capped about 9 Å away from Ser93 by Asp30. The other sides are defined by the mainchain of Gly23/Gly26 and the side chains of Leu92 and Val221 (Figure 2C). It is unclear what functional group the leaving group pocket could accommodate from the unbound FphH structure. Interestingly, the cysteine residue Cys115 that has been shown to be accessible for Coenzyme A modification^38^ is located directly adjacent to Ser93, located similar between the two pockets (Figure 2C). While the Cys side chain is pointing away and is buried by the active site triad, it appears plausible that Cys115 might be able to interact with either binding pockets. Helix 125–140 (Figure 2A) hovers above the active site pockets. For carboxylesterases, this helix has been described as the lid of the active site^39,40^ which folds onto the active site to facilitate catalysis. The structure suggests that the lid is observed in an intermediate to open state, with Ala125 and Gly129 potentially closing the hydrophobic acyl pocket and Tyr133 interacting with the leaving group in the putative closed state (Figure 2C).

### FphH Homologues Have a Conserved Active Site in *Bacillales*

To get a better understanding of how conserved the observations from the FphH structure are, we mapped the 52 sequences listed at Uniprot at 90% identity (Table S2; mostly from different *S. aureus* strains) and the 1304 sequences at 50% identity (from various *Bacillales*) onto the FphH structure (Figure S7). The least amount of conservation is observed in surface exposed areas far from the active site, mostly on helices that connect the core β-strands. Nearly all of the residues discussed in the previous section are highly conserved. The exception is the starting residue of the lid helix Ala125 which differs significantly across sequences. This contrasts with the other lid residues that face active sites Gly129 and Tyr133 and are highly conserved. In the acyl binding pocket, Met192 is the least conserved, within ∼15% of sequences being replaced by other hydrophobic amino acids. In the leaving group pocket, Val221 is the least conserved, being replaced by mostly other hydrophobic amino acids in ∼10% of sequences. Cys115 is also highly conserved, with ∼10% of sequences containing a Ser at this position.

### FphH Structure Compared to Previously Characterized Carboxylesterase Homologues

We performed 3D structure searches using DALI^41^ to identify previously determined structure homologues of FphH (Table S3). Four structures were identified with sequence similarities above 50%. Two of them are unpublished, but the other two have been characterized in detail. Est30 from *Geobacillus stearothermophilus* (PDB ID 1TQH, 1R1D) at 58% sequence identity to FphH was described in 2004 to represent a new subclass of microbial lipases and carboxylesterases.^22^ In 2012, BLA28 from *Bacillus licheniformis* (PDB ID 6NKG) was also classified as a novel family type at 54% sequence identity to FphH (and 70% to Est30). Structure comparison between FphH, Est30 and BL28 indicates that these are closely related homologues with an RMSD of 0.66 Å across 204 Cα pairs and 0.74 Å across 209 pairs, respectively. A major fold difference is in the lid region of residues 115–181 with FphH helices folding distinctly different from the other two. Between these three enzymes, all active site residues that were discussed earlier are conserved, with minor exceptions that FphH Leu168 is an Ile in both Est30/BL28, Ala151 is a Thr in both Est30/BL28 and FphH Val221 is an Ala in just BL28 (Figure S8A). Although the lid folds slightly differently between the structures, the conserved Tyr133 of FphH is exactly matched by Est30/BL28. Notably Est30^24^ was described to be catalytically active as a dimer in solution, and a dimer interface can be see across the symmetry axis with the terminal β-strand running perfectly antiparallel with its symmetry mate in the crystal lattice (Figure S8B). This crystal contact is different for FphH where only the first residue of this β-strand runs antiparallel with its symmetry mate. BL28 adopts a very different crystal stacking (Figure S8B), and it has not been mentioned what oligomeric state BL28 adopts in solution.^26^ While all residues of this β-strand (210–215 FphH numbering) are conserved, the adjacent residues are not. Comparing FphH and Est30 Asp209 is changed to a Val and Ser216 to Glu, which might explain the lack of a clear dimer interface in the crystal structure of FphH. Est30 (1TQH) also contains a covalently bound propyl acetate to the active site Ser which was characterized as a reaction intermediate, while it points toward the leaving group binding pocket in the crystal structure, it was mentioned that this group would fit well into the acyl binding pocket.^22^

### FphH Is a Carboxylesterase with a Broad Substrate Range That Can Be Inhibited by an Oxadiazolone Compound

**Figure 3:**
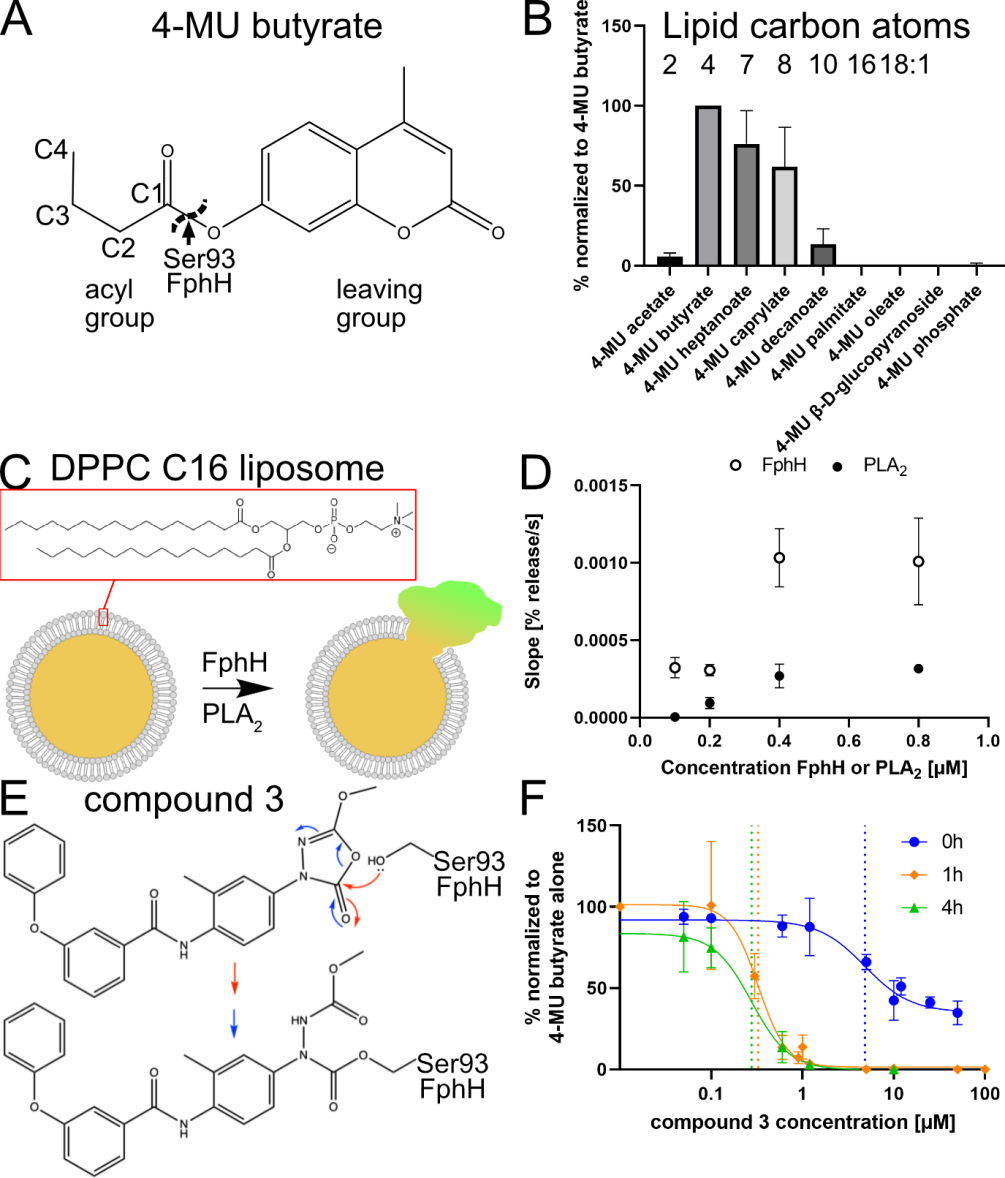
FphH activity assays. A) Illustration of preferred model substrate 4-methylumbelliferyl (MU) butyrate (four carbon atoms C1–C4) cleaved by FphH-Ser93 into an acyl and a leaving group. B) Assessment of the substrate specificity profile using a library of 4-MU based fluorogenic substrates. The graph shows the initial rate for each substrate as relative fluorescence units normalized to the highest rate, which was always 4-MU butyrate. Carbon atoms in the unbranched lipid chain of the model substrate indicated. C) Illustration of DPPC (C16) liposome cleavage by FphH or PLA_2_ to release enclosed fluorescent dye. D) Speed of liposome cleavage observed via dye release dependent on FphH or PLA_2_ enzyme concentration. E) Proposed covalent inhibition mode of compound 3, adopted from Bakker et al.^28^ F) Determination of the half maximal inhibitory concentration (IC50) of compound 3 using 4-MU butyrate as the substrate by FphH. Different preincubation times, 0, 1, and 4 h, of FphH with compound 3 illustrated resulting in IC50 of 4.8, 0.33, and 0.28 μM, respectively.

To uncover how the active site architecture influences enzymatic function, we next delineated the substrate specificity profile of FphH. We tested the substrate preference of FphH using a panel of commercially available 4-methylumbelliferyl (4-MU)-based fluorogenic substrates (Figure 3A,B, *n* = 14 except for inactive 4MU-β-d-glucopyranoside and 4MU-phosphate *n* = 9). FphH could cleave a range of artificial substrates containing unbranched lipids with carbon chain lengths ranging from C2–C10 with the highest activity for the C4 butyrate substrate, followed by C7 and C9. Very little activity was observed for the C2 and C10 substrates, indicating a clear preference of FphH for intermediate chain-length lipids. Such model substrates give only an indication of the physiological substrate but are frequently used in the literature to characterize carboxylesterases/lipases. We performed an extensive literature search to identify 13 homologues with over 30% sequence identity that have been functionally characterized using similar model-substrates (Table S4). The characterized homologues, including Est30 from *Geobacillus*,^19,20,24,25,42^ which showed the highest degree of sequence identity at 58% to FphH, had either the highest activity toward C2 with reduced rates for longer chain substrates or a similar profile to FphH with activities for C2–C10 or slightly longer chains, with preference for ∼C4–C8 (Table S4). Interestingly, the substrate profile of FphH matched very well with that observed for LmH from *Listeria monocytogenes* (52% identity),^27^ with a phylogenetic analysis of these 13 homologues indicating that FphH and LmH are closer related to each other than to *Bacillus* homologues (Figure S9).

Activity against artificial water-soluble short acyl chain esters without activity against long acyl chains categorizes FphH as an esterase/carboxylesterase (EC 3.1.1.1).^43^ We wanted to test if FphH could act as a lipase (EC 3.1.1.3)^43^ using a substrate that more closely resembles a naturally occurring long chain triacylglycerol. We assembled liposomes of a uniform size of about 200 nm from 1,2-dipalmitoyl-*sn*-glycero-3-phosphocholine (DPPC) (Figure S10), that now present an ester bond with a C16 unbranched lipid chain in an environment that resembles a bacterial membrane (Figure 3C). FphH was able to cleave these liposomes, indicated by fluorescent dye release encapsulated in the liposomes, in an enzyme concentration dependent manner (Figure 3D, *n* = 3–6). Cleavage by FphH was about four times faster than by Phospholipase A2 from bovine pancreas (PLA_2_) (Figure 3D, *n* = 3–6) used at the same concentration. PLA_2_ has been studied for over five decades.^44,45^ It often serves as a model enzyme to monitor phospholipase activity against phospholipid liposomes^46^ and in general to study hydrolysis of the β-ester bond of zwitterionic glycerophospholipid with phosphatidylcholine, phosphatidylethanolamine, and their plasmalogen analogues being preferred substrates, while it can also act on phosphatidylinositol and phosphatidylserine.

We also investigated whether FphH has any protease activity. FphH did not have any detectable cleavage activity against 8 model peptides (Table S5) which contain a wide range of peptide bonds, including targets for carboxypeptidases activity.

Next we tested the inhibitory potential of a recently reported putative inhibitor for FphH—the covalent oxadiazole based inhibitor “compound 3” (Figure 3E).^28^ The binding potential of compound 3 toward FphH had previously only been shown for a fluorescent probe version of compound 3 in *S. aureus* lysate. Using 4MU butyrate as the substrate, we determined that compound 3 is indeed a strong inhibitor of FphH with time-dependent IC_50_ due to the covalent inhibition mode. Preincubation of 0 (*n* = 8), 1 (*n* = 4), and 4 h (*n* = 2) with compound 3 resulted in IC50 of 4.8, 0.33, and 0.28 μM, respectively (Figure 3F). Preincubation at room temperature (23 °C) worked for 1 h, with no notable drop in activity for the control without inhibitor between 0 and 1 h. However, after 4 h at room temperature, FphH activity without inhibitor was significantly slower, indicating enzyme stability issues. To still obtain data for a longer incubation period, we repeated the experiment with 4 h preincubation at 4 °C, which did not cause a drop in activity. MALDI-TOF mass spectrometry analysis averaging all proteoforms of FphH incubated with or without compound 3 (417 Da) overnight showed covalent binding of the compound. The mass of FphH alone 28082 ± ∼50 Da (Figure S4A) increased to 28501 ± ∼50 Da and 28912 ± ∼50 Da with compound 3 (Figure S4B). No unbound FphH was observed after incubation with compound 3 and approximately one third of the protein was modified once (28501 Da) and two-thirds twice (28912 Da). Mass spectrometry analysis of tryptic digested inhibitor bound protein identified that as expected, a peptide containing the active site Ser93 was modified (Table S6). The second abundant modified peptide contains Ser45 (and Thr44) and is located on the second β strand.

### FphH Can Hydrolyze Fusidic Acid *in Vitro* but Has No General Protective Effect against This Antibiotic *in Vivo*

**Figure 4:**
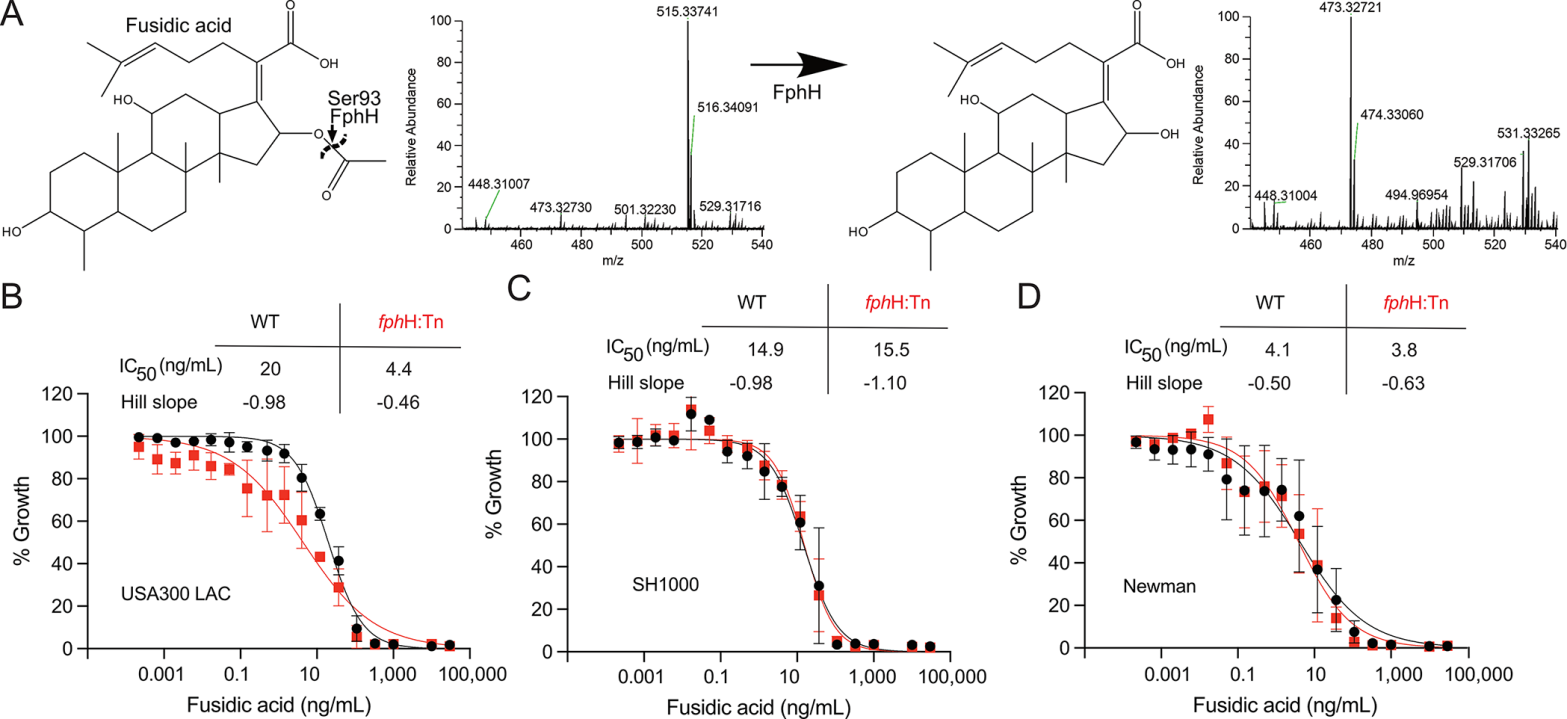
A) Fusidic acid cleavage by FphH identified via mass spectrometry shift of uncleaved 515 Da to cleaved 473 Da. B–D) Growth-inhibitory effects of fusidic acid on *S. aureus* WT and *fph*H:Tn strains. The graphs display the growth inhibitory effect of fusidic acid on the *S. aureus* strains B) USA300 LAC, C) SH1000, and D) Newman and their respective *fph*H:Tn in MH broth. Overnight cultures of the indicated strains were diluted to an OD600 0.01 and incubated in microplates at 37 °C 150 rpm for 16 h in the presence of different concentrations of fusidic acid. The OD600 of the plates was read using a microplate reader and the data normalized to the density of the negative control in the absence of antibiotic. The IC_50_ and Hill slope (h) are indicated for WT and their corresponding *fph*H:Tn. The graphs show means ± SD of three independent biological replicates. The shown nonlinear regression curves were fitted using the “log (inhibitor) vs normalized response–variable slope” equation in GraphPad Prism 9 software.

In a previous study, *fphH* expression was found to be highly upregulated in response to the antibiotic fusidic acid.^29^ This led us to hypothesize that FphH might either be a secondary target of fusidic acid or use fusidic acid as a substrate, potentially inactivating it through hydrolysis. Incubation of fusidic acid with FphH overnight clearly showed cleavage of the acetyl group of the antibiotic via mass spectrometric analysis—a mass change of 515 to 473 Da corresponds exactly to loss of an acetyl group (Figure 4A).

Intrigued by the possibility that FphH may have a protective role against the antibiotic effect of fusidic acid, we compared the susceptibilities of the *fph*H:Tn and WT strains against this antibiotic. Since upregulation of FphH was observed in strain SH1000,^29^ but our available *fph*H:Tn were in strain USA300 (LAC) and Newman, we transferred the transposon insertion in the *fph*H gene into this strain background by phage transduction. Comparative analysis of the dose-dependent growth inhibitory effects to fusidic acid revealed that among the different WT strains, USA300 (LAC) had the lowest susceptibility, as quantified by IC50 determination, followed by SH1000 and Newman (Figure 4B–D). The dose–response curves also differed in steepness: USA300 (LAC) and SH1000 had Hill Slopes (h) around −1.0, while the more susceptible strain, Newman, exhibited a shallower progression with a Hill slope (h) of −0.50.

Compared to its parent strain, the *fphH*:Tn mutant based on the USA300 (LAC) exhibited a roughly 4-fold reduction in IC_50_ concentration, and its dose–response progression was notably shallow (*h* = −0.46) (Figure 4B). In contrast, in strains SH1000 and Newman, inactivation of the *fph*H gene did not have any effect on the susceptibility to fusidic acid (Figure 4C,D).

## Discussion

The clear preference for the butyrate over the acetate chain length indicates a level of specificity that places FphH between our previously characterized *S. aureus* FphB^3^ and FphF^5^ carboxylesterases. Based on the substrate profile, the FphB activity is very narrow with a preference for butyrate and no activity toward acetate, while FphF is more promiscuous across a wider range of chain lengths. Further evidence for functional redundancy between different members of the Fph family and/or the secreted lipases comes from activity-based protein profiling studies where we previously observed that the *fph*H:Tn mutant on strain USA300 (LAC) and Newman background showed elevated activity of the secreted lipases SAL1/SAL2, which are related to FphH and the other Fphs, (but were not renamed, since they had been characterized previously) and/or FphE.^3,4^ Despite evidence for biochemical redundancy on synthetic substrates *in vitro* and compensatory upregulation in cells *in vivo* described above, it must be noted that the subcellular localization of these enzymes is expected to differ. SAL1/2 are secreted, FphB is localized in the cell envelope and FphE is predicted to be localized in the membrane with the hydrolase domain oriented toward the cytosol.^3^ The work presented here reports the absence of putative transmembrane domains in FphH, clearly suggesting cytosolic localization. The ability of FphH to cleave an acetyl group off the antibiotic fusidic acid while also being able to process C16 liposomes makes it difficult to predict the exact physiological substrate and the corresponding biological role.

Fusidic acid is a natural product antibiotic that blocks prokaryotic translation by binding to elongation factor G.^47^ In *S. aureus*, the most common mechanisms of resistance is spontaneous mutations in the *fus*A gene which encode the molecular target EF-G, or through acquisition of genes (*fusB*, *fusC* or *fusD*) leading to protection of the target site, while other mechanisms that, e.g., involve drug permeability also exist.^48,49^ Already in the 1990s^50^ it was shown that fusidic acid-resistant *Streptomyces spp.* can deacetylate fusidic acid through an esterase termed *fus*H. The authors concluded that “*as fusidic acid is used to treat various infections caused by Staphylococcus aureus and other micro-organisms, the possibility that these pathogens can acquire resistance due to the synthesis of a FusH-like activity deserves attention*.” We were prompted to investigate if FphH may carry out such FusH-like activity since a study on the transcriptional response of *S. aureus* SH1000 revealed a 16.8-fold upregulation of the fphH gene (*est*, SACOL0845).^29^ Our data clearly indicate that recombinant FphH can indeed deacetylate and thus inactivate fusidic acid *in vitro*. In the cellular system, however, we found that transposon-based inactivation of *fph*H did not affect the susceptibility to fusidic acid. Only the *fph*H:Tn mutant on USA300 (LAC)-background showed some growth reduction when exposed to a sub-MIC concentration of fusidic acid. These results suggest that either the function of FphH is compensated for by functionally redundant enzymes or that the hydrolytic activity of FphH on fusidic acid *in vitro* is not physiologically relevant in cells.

Of note, a recent study reported the transcriptional response of an *in vitro*-evolved fusidic acid-resistant strain derived from *S. aureus* SH1000.^51^ This strain carried mutations in the *fus*A gene, which encodes EF-G and a putative phage gene. The transcriptional analysis highlighted several significant findings. Aside from the upregulation of capsule genes that likely affect permeability to the drug, there was also upregulation (19- and 11-fold respectively) of both SAL1 and SAL2 (*geh*A and SACOL0390) in the resistant strain. This upregulation might contribute to the hydrolytic inactivation of fusidic acid. Expression levels of *fph*H, were not different from those observed in the parent strain. Concurring with the results of this study, it appears likely that the upregulation in *fphH* in response to fusidic acid^29^ reflects the stress response induced by the antibiotic. This study also concluded that the functional response to fusidic acid (which effectively leads to a block in translation) is similar to cold shock stress-response and the stringent response.^29^

A potential role in stress responses could also explain most of the deficiencies in growth under nutrient-deprived conditions in stationary phase, biofilm formation, and intracellular survival that we observed when phenotypically analyzing *fph*H:Tn mutant strains. These data must be interpreted with caution, since we did not perform complementation studies and cannot exclude that secondary mutations may contribute to these phenotypes. The fact that compensatory upregulation of FphE activity in the *fph*H:Tn mutant in strain Newman is particularly pronounced in stationary phase^4^ supports the notion that indeed the hydrolytic activity of FphH is functionally important under these conditions. The strict conservation of the *sec*G-*fph*H-*rnr*-*smp*B-*ssr*A gene cluster not only highlights its putative relevance but also suggests that the genes contribute to the same functional processes,^11^ even though expression of these genes might also be regulated individually.^11,29,52^ Our results support this notion since both *fph*H:Tn and *rnr*:Tn mutant strains both showed a deficiency in biofilm formation but differed in other phenotypes such as survival in THP-1 cells. Various gene expression profiling studies in *B. subtilis* have suggested a primary role for this gene cluster in stress responses,^13−15^ which is further supported by proteomic analysis done in *S. aureus* COL, that demonstrated that sigma factor deltaS, involved in antibiotic and stress responses, positively influences *fph*H (SACOL0845).^53^ In contrast, in strain N315, expression of *fph*H (SA0734) (as well as *fph*B (SA2323), *fph*D (SA1990)) were found to be negatively regulated by SarS.^54^ In addition, in *L. monocytogenes*, the FphH homologue Q8Y4I9 (Lmo2450) but no other member of the *secG-yvaK-rnr-smpB* cluster was repressed in 1,2-propanediol and ethanolamine induced stress conditions.^52^

Two lines of evidence point toward a physiological role of FphH related to ribosome malfunction: (i) Its upregulation in response to the ribosome-binding fusidic acid and the minor phenotype we observed in the dose–response curve of a USA300 (LAC)-based *fph*H mutant. (ii) The location of the gene in a conserved gene cluster alongside genes with a demonstrated role in the ribosome rescue pathway.^11^ What role could a hydrolase have in these ribosome-related processes? In the ribosome rescue system^55^ stalled ribosomes are rescued by the function of *smp*B/*ssr*A where the tmRNA (*ssr*A) replaces *e.g*. truncated nonstop mRNA from the ribosome and leads C-terminal attachment of a peptide tag, that will induce degradation by ATP-dependent proteases such as Lon, FtsH or the ClpXP complex. It is common that proteases retain carboxylesterase activity, and it is plausible that, as of yet, unidentified proteases further contribute to this proteolytic degradation, but it seems to be an unlikely role for FphH since we did not detect any protease activity of FphH against a panel of synthetic fluorogenic protease substrates. A more suitable substrate for FphH in accumulating peptidyl tRNAs upon ribosome blockade is the ester linkage between peptide and tRNA. Unless the *trans*-translation system is active to induce completion of translation and target the resulting protein for degradation, the peptidyl tRNAs need to be cleaved and recycled in order to prevent accumulation of nonfunctional proteins and to recycle its components. *S. aureus* has a homologue of the well characterized peptidyl tRNA hydrolase (*pth*) of *E. coli*,^56^ but it appears possible that *S. aureus* and other *Bacillales* contain additional genes with similar function.

The displaced faulty mRNA should also be degraded, and Ribonuclease R (in the gene cluster gene *rnr*) is a likely candidate contributing to its hydrolysis.^57^ Ribonuclease R is particularly effective cleaving RNAs with secondary structures, including tRNA and rRNA,^58^ but it also contributes to cleaving mRNA,^59^ which may induce *trans*-translation. In *E. coli*, it has been shown that the stability of Ribonuclease R can be modified by acetylation.^60^ A recent proteomic study has revealed that Ribonuclease R (VacB/RNase II family exoribonuclease, AID39289.1), SmpB (SSrA-binding protein AID39290.1) as well as FphH (carboxylesterase, AID39288.1) have multiple acetylation sites in *S. aureus*, where SmpB also features one succinylation site.^61^ Thus, the hydrolytic removal of these post-translational modifications on Ribonuclease R and SmpB is a hypothetical function for FphH/YvaK that might link this esterase to the other members in the gene cluster. Even though FphH demonstrated a preference for butyrate over acetate chain lengths across our panel of synthetic fluorogenic substrates, our overall structure–function characterization clearly indicates that FphH is able to deacetylate substrates like fusidic acid.

It was previously suggested that *trans*-translation and ribosome recycling could be a relevant source for amino acids under starvation.^55^ In this light, one hypothetical scenario that would link the phenotypes we observed with the *fph*H:Tn mutants, its biochemical function as a carboxylesterase, and gene expression profiling results on the expression and function of the other genes in the cluster would be the following: FphH is expressed under stress conditions, including starvation that the cells experience in stationary phase. FphH could deacetylate Ribonuclease R leading to its stabilization, and in turn, Ribonuclease R might cleave mRNA leading to ribosome stalling and induction of *trans*-translation, resulting in a stop of translation and amino acid recycling. This hypothesis is currently under investigation.

It is of course possible that FphH acts on substrates nonrelated to the ribosome function. Our structure–function characterization indicates that FphH will cleave off a C2 to up to a C16 hydrophobic group from a substrate or directly act on a membrane lipid. In addition to the already discussed possibility of post-translational modification on proteins, a small hydrophobic group, e.g. a lipid chain could be located on small molecules, similar to a serine hydrolase involved *S. aureus* menaquinone synthesis.^62^ On the other hand our liposome cleavage studies also allow for the possibility that FphH might play a role in membrane assembly and maintenance, especially in biofilms

Beyond the role of FphH in the *S. aureus* life cycle, there is also interest in the industrial application of esterases/lipases such as FphH. The majority of FphH homologue characterizations in the literature have focused on potential industrial applications of these lipases without investigating the biological role. The limited stability of FphH observed in our study when incubated at RT for 4h, makes FphH less suitable for industrial applications. In contrast, many homologues from thermostable bacteria have been characterized,^63^ and their thermostability has been linked to the lid or cap region of these proteins^63^ which differs between FphH and the homologues from thermophilic bacteria. This also demonstrates that FphH must have evolved to perform a different role in *S. aureus* compared to closely related thermostable bacteria. This difference in lid fold is also notably observed when comparing the experimentally determined FphH structure to the Alphafold^36^ prediction. The Alphafold prediction appears to be heavily based on the thermostable homologue structures, and this observation highlights the continuous need to determine protein structures, even for enzymes where the overall fold is well-known. Minor changes in the active site pockets, combined with differences in the fold of the lid and oligomeric state, might explain different preferences between FphH and thermostable homologues. Especially the lid architecture has been mentioned to determine activity toward certain substrates for this enzyme fold.^64^ Overall, it appears that FphH is more closely related to the homologue from the pathogen *Listeria monocytogenes*(27) than homologues from thermophilic *Bacillus* bacteria.

That FphH can be targeted during the *S. aureus* life cycle by small molecules has been demonstrated.^3,4,28^ Here we show that one of these small molecules, an oxadiazolone is indeed a strong inhibitor of FphH that covalently and therefore permanently binds to the active site serine residue, the proposed reaction mechanism of oxidazolones^28^ with serine hydrolases also appears to be confirmed by this study. Based on our atomic characterization of FphH, future structural studies of FphH in complex with small molecules bound to the active site could aid in the development of compounds that specifically target FphH. Such FphH-specific inhibitors could play important roles in further dissecting the molecular function of FphH, as they would enable temporally controlled inactivation of FphH, which could circumvent functional compensation issues that may be displayed by mutant strains. In addition, inhibition of FphH and thus putative interference with *S. aureus* stress responses could attenuate important infection-related processes and may have therapeutic potential.

## Methods

### FphH Sequence Database Searches

For all 90% identical sequences to Uniprot^10^ entry of FphH Q2G025, a list with all gene identifiers and literature mentions of these identifiers in Google Scholar was compiled. Within the 50% identical sequences, the genes from the most well studied bacterial strains were also examined via Google Scholar. In depth literature searches were performed to identify all *in vitro* characterized homologues of FphH, sequence information was gathered from Uniprot,^10^ alignments were created using Clustal Omega^65^ and phylogenetic trees using IcyTree.^66^

### FphH Cloning, Expression and Purification

The full length *fph*H (currently annotated as *est*, gene loci SAUSA300_ 0763) was amplified from the *S. aureus* USA300 genome using primers CAG GGA CCC GGT ATG CAG ATA AAA TTA CCA AA and CGA GGA GAA GCC CGG TTA TTC TGA CCA GTC TAA TGA CT that introduced overhangs for ligation-independent cloning.^67^ The PCR product was gel-purified and cloned into modified pET28a-LIC vectors incorporating an N-terminal His_6_-tag and a 3C protease cleavage site.

*E. coli* BL21(DE3) cells in 1 L cultures (1X Luria–Bertani with 50 μg/mL kanamycin) at 37 °C and 200 rpm shaking were grown until OD_600_ reached 0.6. Cultures were induced with 0.2 mM Isopropyl β- d-1-thiogalactopyranoside and grown overnight at 18 °C and 200 rpm shaking. Bacterial pellets were harvested via centrifugation, suspended in 20 mM Tris pH 8.0, 300 mM NaCl and stored at −20 °C.

For purification, thawed suspended pellets were incubated for 30 min on ice with ∼20 μg/mL lysozyme. Cells were lysed via sonication (Sonifier Heat Systems Ultrasonics). FphH protein was initially purified by Ni^2+^ affinity chromatography (HIS-Select resin, Sigma-Aldrich) using an elution buffer containing 50 mM Tris pH 8.0, 300 mM NaCl, 300 mM Imidazole, 10% (v/v) glycerol and 10% (w/v) sucrose. Elution fractions were incubated with 3C protease and 2 mM DTT overnight at 4 °C. FphH was further purified by anion exchange (RESOURCE Q, 10 mM HEPES pH 7.5/7.6, gradient 10 to 1000 mM NaCl) and finally by size-exclusion chromatography (10 mM HEPES pH 7.5/7.6, 100 mM NaCl) using a Superdex 75 Increase column (GE Life Sciences). Purified protein was either used directly for crystal drops or snap frozen in liquid nitrogen.

### FphH Enzymatic Activity Assays

An enzymatic activity assay of purified FphH protein was carried out using a series of 4-MU-based fluorogenic substrates with various unbranched lipid carbon chain lengths or a phosphate or sugar group to determine the substrate specificity. Using a Greiner 96-well, flat-bottomed plate, 30 μL of reaction volume contained 4.2 nM FphH and 10 μM substrate in 1× PBS pH 7.4/0.01% TritonX-100 buffer. The fluorescence (λ_ex_ = 365 nm and λ_em_ = 455 nm) was measured at 37 °C at 30-s intervals on a CLARIOstar instrument for 60 min. The initial rate of the reactions was determined using the GraphPad prism software and normalized to the 4-MU-Butyrate rate as this one was the highest rate for each assay. No significant rates of FphH or the substrate alone were observed as controls. Using two different FphH preparations, 14 replicates via 7 distinct setups for each lipid substrate and 9 replicates (4 setups) for the phosphate and sugar substrate were measured to determine the substrate preference.

To determine the IC_50_ of Compound 3, 11 different concentrations of Compound 3 (0.1 to 100 μM) were incubated with FphH at room temperature (23 °C) for 0 and 1 h. For the 4 h experiment, incubation was changed to 4 °C. The initial rate of 4-MU-Butyrate was determined as described above and normalized to a control (0 μM Compound 3). The IC_50_ was determined by using the GraphPad prism software. Compound 3 was synthesized as previously reported.^28^ The potential inhibitory effect of fusidic acid was measured similarly as described for compound 3 using 5 different concentrations (0.1 to 100 μM) and one h of preincubation, with no inhibition observed.

Details about the FphH triggered fluorescent dye release from 1,2-dipalmitoyl-*sn*-glycero-3-phosphocholine (C16) liposomes at 37 °C in pH 1× PBS/0.01% TritonX-100 pH 7.4 can be found in the Supporting Information.

### MALDI TOF Intact Protein Measurement

For compound 3 covalent binding to FphH, 0 or 1 mM compound 3 was incubated with FphH (42 μM) in parallel in 90% 1X PBS pH 7.4, 10% DMSO overnight at 22 °C. The intact FphH protein measurement with or without compound 3 was carried out on a MALDI-TOF 4800 mass spectrometer (ABSciex, USA). FphH was mixed in a ratio of 1:1 with α-cyano-4-hydroxycinnamic acid (CHCA) before being spotted on a MALDI plate. The sample was measured in linear positive ion mode. The resulting data were interpreted using free msMass software (www.mmass.org). (LC)-coupled electrospray high resolution mass spectrometry was unable to obtain a clean signal of the intact FphH protein, indicating many differentially modified forms of the same protein.

### FphH-Compound 3 Characterization by LC-Tandem Mass Spectrometry

Compound 3 bound FphH was digested with trypsin (Promega, USA) in a ratio of 1 part trypsin to 20 part FphH. The trypitically cleaved peptides were chromatographically separated on a 20 cm emitter-tip column (75 um ID fused silca tubing (CoAnn Technologies, USA) packed with 3 μM C-18 Luna material (Phenomenex, USA)) on an Ultimate 3000 uHPLC (Thermo Scientific, USA). The peptides were eluted from the column using a short 20 min gradient from 5 to 60% acetonitrile. Peptides were measured by an LTQ Orbitrap XL (Thermo Scientific, USA) mass spectrometer at a resolution of 60000 @ *m*/*z* 400. The 8 strongest ms1 precursors were selected for collision induced dissociation (CID) fragmentation in the ion-trap. The resulting data was searched with the Sequest search engine using the Proteome Discoverer pipeline (version 2.5) (Thermo Scientific, USA). The mass adduct of compound 3–417.132 was allowed as a dynamic modification on serine and threonine. The data was queried with semitrypsin option on an in-house fasta protein database containing the sequence of FphH.

### Fusidic Acid Cleavage Detection by LC-Mass Spectrometry

For cleavage of fusidic acid by FphH, 0 or 1 mM fusidic acid was incubated with FphH (42 μM) in parallel in 1× PBS pH 7.4 overnight at 22 °C. The mass of the intact and the cleaved form of fusidic acid was measured on an Obritrap Exploris 240 mass spectrometer (Thermo Scientific, USA) coupled to a Vanquish Flex uHPLC (Thermo Scientific, USA). The compounds were subjected to reverse phase chromatography on an Aeris 1.7 μm PEPTIDE XB-C18 100 Å, LC Column 150 × 2.1 mm (Phenomenex, USA) on a short gradient of 5 to 100% acetonitrile. Compounds were measured at the resolution of 240000 in positive ion mode. The resulting data were manually interpreted in the FreeStyle software (Thermo Scientific, USA).

### FphH Crystallization

FphH was broadly screened for crystallization, resulting in multiple hits with details given in supporting Table S2. For the unbound FphH structure presented here, 0.15 μL of 9.1 mg/mL FphH (10 mM HEPES pH 7.6, 100 mM NaCl) was mixed with 0.3 μL of reservoir solution. The sitting drop reservoir contained 200 mM Calcium acetate hydrate, 100 mM Tris pH 7.5, 10% w/v PEG 8000 and 10% w/v PEG 1000. Crystals appeared within 1 day at 16 °C and grew until day 12.5. Crystals were soaked for ∼15 s in 75% reservoir solution and 25% glycerol, 75% reservoir prior to freezing in liquid nitrogen.

### FphH Data Collection, Processing, Refinement, Deposition, and Analysis

X-ray diffraction data were collected at the Australian synchrotron MX2^68^ beamline. Data sets were processed with XDS,^69^ merging and scaling were performed using AIMLESS.^70^ Phases were solved with Phenix Phaser molecular replacement^71^ using a model form Alphafold^36^ via Uniprot^10^ for FphH *S. aureus* strain USA300 A0A0H2XJL0. Model building and refinement were conducted in COOT^72^ and Phenix.^73^ The final structure was deposited to the worldwide protein databank^74^ PDB ID 8FTP. Statistics for the data sets are listed in Tables S7 and S8. Structure figures, analysis and alignments were created with UCSF Chimera.^75^ A protein structure similarity search was conducted using using the determined FphH structure were performed using the Dali Web server^41^ and the worldwide protein databank^74^ (Table S3).

### Bacterial Strains and Handling

The *Staphylococcus aureus* strains USA300 (LAC), SH1000, Newman and JE2 were used as the wild-type (WT) strains. To investigate the role of the *fph*H and *rnr* in in these strain backgrounds, the transposon mutant designated as *fph*H:Tn and *rnr*:Tn respectively were derived from the respective WT strain. To prepare the bacterial strains for experimentation, all *S. aureus strains* were subcultured from cryogenic storage onto Tryptic Soy Agar (TSA) plates and incubated overnight (16 h) at 37 °C. TSA is a commonly used medium for bacterial growth. After incubation, the colonies were picked and transferred into Tryptic Soy Broth without antibiotics and routinely maintained at 37 °C for further experimentation.

### Growth Curve

The start-up cultures of the *S. aureus* strains were grown in Tryptic Soy Broth (TSB) overnight at 37 °C. Briefly, the cultures were normalized to an OD600 of 0.1 and 2 μL inoculated into 150 μL of TSB in a 96-well microtiter plate. Then, the plates were incubated at 37 °C in a Microplate Reader Synergy HI with periodic 10 s shaking before reading the absorbance measurement at A600 nm at 15 min intervals for 48 h.

### THP-1 Cell Infection and Metabolic Activity Assay

Human derived THP-1 cell lines were used to investigate the ability of the strains to infect macrophages. The differentiated THP-1 cell lines were seeded into 24-well plates at a density of 1 x10^6^ cells/mL in RPMI-1640 (supplemented with 10% v/v heat-inactivated fetal bovine serum (FBS), 1% Penicillin/Streptomycin and 0.05 mM 2-mercaptoethanol) (Sigma) and incubated for 24 h at 37 °C, 5% CO_2_. Overnight cultures of the *S. aureus* strains in Tryptic Soy broth (TSB) were subcultured into fresh TSB and grown to an OD600 of 1. The bacterial density was adjusted to 1 × 10^7^ CFU/mL in RPMI-1640 without Penicillin/Streptomycin) and added to each well seeded with 1 × 10^6^ cells/mL of THP-1 cells at a MOI of 10. The cells were infected for 45 min, washed with PBS and incubated for 30 min in RPMI-1640 (supplemented with 100 mg/mL gentamicin and 10% heat-inactivated FBS) to eliminate extracellular bacteria. The cells were washed twice with PBS and lysed with PBS containing 0.2% v/v triton X-100 (Sigma). To determine the number of viable bacteria, the lysates were serially diluted in PBS, plated on Mueller Hinton Agar plates, and colonies were counted after incubating at 37 °C for 24 h.

The test for THP-1 cell viability following infection with the *S. aureus* strains was mitochondrial metabolic activity using the tetrazolium dye, MTT (3-[4,5-dimethylthiazol-2-yl]-2,5 diphenyl tetrazolium bromide). We performed the MTT assay to measure the effect of the strains on the THP-1 cells’ metabolic activity, which is indicative of the cytotoxic effect of the bacteria on the THP-1 cell or their ability to kill the THP-1 cells (viability). The THP-1 cells were seeded at 5000 cells per well in a 96-well plate. Overnight cultures of *S. aureus* strains grown in TSB at 37 °C were subcultured into fresh TSB, grown to OD600 of 1 and diluted to 1 × 10^7^ CFU/mL in RPMI-1640. The THP-1 cells were infected at an MOI of 10 for 1 h. Following the infection, the cells were washed with PBS and tetrazolium dye was added at a final concentration of 5 mg/mL. The plates were incubated for 2 h at 37 °C and 5% CO_2_. Then, the media were aspirated from each well, and the purple formazan crystals were dissolved with DMSO for 1 h at 37 °C. The absorbance proportional to the viability of the THP-1 cells was measured at A570 nm.

### Protein A Assay

Costar 96-well ELISA plates were coated with 10 μg of antiprotein A antibodies (ACRIS) in PBS overnight at 4 °C. After overnight coating, the plates were washed three times with PBS-T (100 μL PBS containing 0.05%v/v Tween) and air-dried at room temperature for 1 h. Additionally, the plates were blocked with 1% Bovine Serum Albumin (BSA) for 2 h at 37 °C. Overnight cultures of *S. aureus* strains grown in TSB at 37 °C and 200 rpm were subcultured into fresh TSB and grown to OD600 of 1.0. Briefly, 100 μL of the bacteria were added to the wells and incubated at 37 °C for 1 h. Then, the plates were washed two times with 200 μL of PBS-T (PBS containing 0.05% Tween), fixed with 100 μL of 4% paraformaldehyde for 20 min at room temperature, washed two times with ddH_2_O and air-dried. The adhered bacterial cells in the wells were stained with 150 μL of 0.1% w/v crystal violet and incubated at room temperature for 5 min. The plates were then washed two times with ddH_2_O, and the crystal violet was solubilized with 200 μL of 30% v/v acetic acid at room temperature for 15 min. The absorbance was measured at A595 nm.

### Biofilm Assay

*S. aureus* strains grown overnight at 37° in TSB were subcultured into fresh TSB and grown to an OD600 0.5. Briefly, 1 μL of the cultures was inoculated into Nunclon 96-well plates (Thermoscientific) containing 200 μL of TSB. The plates were incubated for 16 h at 37 °C without agitation. First, the planktonic cells were washed off using low-pressure running water, and the plates were air-dried before staining with 200 μL Crystal violet (0.1% w/v)(Sigma-Aldrich) for 10 min at room temperature. Next, the staining solution was rinsed off, and the dye was solubilized using 200 μL dimethyl sulfoxide (DMSO)(Sigma-Aldrich) for 10 min at room temperature. The absorbance was measured at A590 nm using a microplate reader.

### Agar Diffusion Assays

Production of nucleases and hemolysis by the *S. aureus* were conducted using agar diffusion assays. Overnight cultures of *S. aureus* strains grown in TSB at 37 °C were subcultured in fresh TSB to OD600 = 0.5. Briefly, 10 μL of the cultures were spot-inoculated on the bioassay plates and incubated overnight at 37 °C. Nuclease production was quantified using the DNase agar plates (Oxoid). Following overnight incubation, the plates were flooded with 1 M Hydrochloric acid (VWR) for 10 min at room temperature to develop a better contrast illustrating the zone of DNA clearance. To determine the level of protease production, we measured the diameter of the zone of clearance around the colonies from four different points and calculated the average diameter. Hemolysis of red blood cells was quantified using blood agar plates. Following overnight incubation, plates were kept at 4 °C overnight before measuring the diameter of the zone of clearance around the colonies. In each agar diffusion assay, the agar plate was inoculated with both the WT and the *fph*H mutant. Subsequently, we measured the diameters of their respective zones of clearance. The zone of clearance for the *fph*H mutant was then expressed as a percentage of the WT, with the WT’s zone normalized to 100%.

### Fusidic Acid Susceptibility Testing

Susceptibility of different *S. aureus* strains to fusidic acid was determined using the broth microdilution method. The starting stock solution of fusidic acid was prepared at a concentration of 30 mg/mL, and 16 3-fold dilutions were prepared in the 96-well microplates. The start-up cultures of the *S. aureus* strains were incubated in MH media for 16 h at 37 °C and 200 rpm. The cultures were diluted in fresh MH media to OD600 0.01 per well together with the appropriate concentration of the fusidic acid. Next, the plates were incubated at 37 °C at 150 rpm for 16 h, before OD600 was measured in a microplate reader. For determination of IC_50_ concentrations data were normalized and nonlinear regression analysis (“log (inhibitor) vs normalized response–variable slope” equation) was performed in Prism 9 (GraphPad software LLC).

## Data Availability

The data sets generated and analyzed during the current study are available in the worldwide Protein Data Bank under PDB ID 8FTP (FphH unbound). Authors will release the atomic coordinates and experimental data upon article publication.

## Supporting Information Available

Contains additional methods details, supporting Tables S1–S8 and supporting Figures S1–S11 (PDF)

## Abbreviations

Fph: fluorophosphonate-binding hydrolase
