# Supplementary material for: Biochemical and Cellular Characterization of the Function of Fluorophosphonate-Binding Hydrolase H (FphH) in Staphylococcus aureus Support a Role in Bacterial Stress Response
Source: ACS Infect Dis. 2023 Oct 12;9(11):2119–32. doi: 10.1021/acsinfecdis.3c00246 (PMC10644348; doi:10.1021/acsinfecdis.3c00246)
Supplement: Supplementary file 1 — id3c00246_si_001.pdf [file id3c00246_si_001.pdf]

Supporting information

Biochemical and Cellular Characterization of the Function of Fluorophosphonate-Binding Hydrolase H (FphH) in *Staphylococcus aureus* Support a Role in Bacterial Stress Response.

Matthias Fellner[1]*, Annabel Walsh[1], Stephen Dela Ahator[2], Nadia Aftab[2], Ben Sutherland[3], Eng W. Tan[3], Alexander T. Bakker[4], Nathaniel I. Martin[5], Mario van der Stelt[4] and Christian S. Lentz[2]*

[1]Biochemistry Department, School of Biomedical Sciences, University of Otago, Dunedin 9054, New Zealand

[2]Research Group for Host-Microbe Interactions, Department of Medical Biology and Centre for New Antibacterial Strategies (CANS), UiT – The Arctic University of Norway, 9037 Tromsø, Norway

[3]Department of Chemistry, Division of Sciences, University of Otago, Dunedin 9054, New Zealand

[4]Department of Molecular Physiology, Leiden Institute of Chemistry, Leiden University, 2333 CC Leiden, The Netherlands

[5]Biological Chemistry Group, Institute of Biology Leiden, Leiden University; Leiden 2333 BE, The Netherlands

*To whom correspondence should be addressed: Matthias Fellner: Department of Biochemistry, School of Biomedical Sciences, University of Otago, PO Box 56, Dunedin 9054, New Zealand; matthias.fellner@otago.ac.nz and Christian Lentz: Department of Medical Biology, UiT – The Arctic University of Norway, 9037 Tromsø, Norway; christian.s.lentz@uit.no

Table of contents:

- Detailed methods section for enzyme triggered fluorescent dye release from liposomes.
    - Equation 1: % CF Released.
- Table S1: Crystallization conditions of FphH.
- Table S2: FphH Q2G025 90% or higher sequence identical proteins listed in UniProt.
- Table S3: Protein structures similar to FphH.
- Table S4: *In vitro* characterized lipases/carboxylesterases similar to FphH.
- Table S5. Model peptide substrates IQ1-IQ8 that did not have any cleavage activity by FphH.
- Table S6. Tryptic digested compound 3 bound FphH mass spectrometry (MSMS) analysis.
- Table S7: FphH structure data collection and processing.

- Table S8: FphH structure solution and refinement.
- Figure S1. Functional characterization of *rnr*:Tn strain.
- Figure S2: Gel-filtration chromatogram of FphH.
- Figure S3: SDS-PAGE of FphH.
- Figure S4: MALDI-TOF intact protein measurement.
- Figure S5: Calcium binding in the FphH crystal.
- Figure S6: FphH B-factor analysis.
- Figure S7: FphH structural conservation.
- Figure S8: Comparison of FphH homologs.
- Figure S9: Phylogenetic tree of homologous family of *S. aureus* FphH.
- Figure S10: Dynamic light scattering (DLS) measurements of liposome batches.
- Figure S11: PCR-validation of *fph*H:Tn strains.

Detailed methods section for enzyme triggered fluorescent dye release from liposomes.

Materials

1,2-dipalmitoyl-sn-glycero-3-phosphocholine (DPPC) was purchased from Lipoid. Phospholipase A2 from bovine pancreas (PLA_2), and Triton-X-100 were purchased from Sigma Aldrich. 5(6)-Carboxyfluorescein (CF) was purchased from Molekula. All chemicals were used without further purification.

Preparation of dye loaded liposomes

Liposomes were prepared using the thin film rehydration method. 10 mg of DPPC was dissolved in chloroform, the solvent was removed in vacuo and dried at 10 mbar for 30 minutes to leave a thin lipid film. The dry lipid film was rehydrated with a CF solution (1 mL, 100 mM in pH 9 PBS), followed by alternating 1 min of vortexing and 10 min of sonication three times. The resultant suspension was extruded through two stacked 200 nm pore sized polycarbonate membranes 15 times in an Avanti® Polar lipids mini extruder preheated to 60 °C to achieve size controlled, unilamellar vesicles. Non-encapsulated dye was removed from the suspension by dialysis in pH 7.4 PBS (2 L total). Hydrodynamic size and size distribution of the liposomes were characterized by DLS (Malvern Panalytical Zetasizer Nano ZS). All measurements were repeated in triplicate at 25 °C.

Enzyme triggered release from liposomes

Release studies were performed by measuring the change of fluorescent intensity of the self-quenching fluorophore CF. Enzyme liposome samples were prepared from FphH or PLA_2 solution (at an enzyme concentration of 0 µM, 0.1 µM, 0.2 µM, 0.4 µM, 0.8 µM and 1.6 µM in pH 7.4 PBS) with CF loaded Liposome suspension (2.5 % v/v). Fluorescence intensity measurements were made on a BMG LabTech CLARIOStar microplate reader. Samples were incubated at 37 °C and measurements were made every 20 seconds using an excitation wavelength of 470 nm and emission wavelength of 520 nm. To obtain the fluorescence intensity at 100% dye release, lysed liposome samples were prepared from FphH or PLA_2 solution (at an enzyme concentration of 0 µM, 0.1 µM, 0.2 µM, 0.4 µM, 0.8 µM and 1.6 µM in

pH 7.4 PBS), Triton-X-100 (0.75% v/v), and CF loaded Liposome suspension (2.5% v/v) followed by quantification of fluorescence intensity. Intensity measurements were repeated in triplicate.

The percentage release of CF from the liposomes was calculated from the equation (1).

$$\%\ CF\ Released = \frac{I_t - I_0}{I_{max} - I_0} \times 100 \quad (1)$$

Where I_0 is the initial fluorescent intensity at t = 0, and I_{max} is the intensity obtained from liposomes lysed with Triton-X-100. All measurements were repeated with three separately prepared batches of CF loaded liposome suspensions. All data was corrected by background %CF released of a sample only containing pH 7.4 PBS.

Table S1: Crystallization conditions of FphH.

Highest diffracting crystal in Å, with dataset presented in this publication originating from crystal form 1.

(Å)	Compound 1	Compound 2	Compound 3
**Crystal form 1***			
1.37	0.2 M Calcium acetate hydrate	0.1 M Tris pH 7.5	10 % w/v PEG 8000,<br>10 % w/v PEG 1000
1.65	0.2 M Calcium acetate hydrate	0.1 M Tris pH 8.5	10 % w/v PEG 8000,<br>10 % w/v PEG 1000
1.70	0.2 M Calcium acetate hydrate	0.1 M MES pH 6.5	15 % w/v PEG 4000
1.83	0.2 M Calcium acetate hydrate	0.1 M MES pH 7.5	10 % w/v PEG 8000,<br>10 % w/v PEG 1000
**Crystal form 2****			
1.80	0.2 M Calcium acetate hydrate	0.1 M Tris pH 8.5	25 % w/v PEG 2000 MME
1.84	0.2 M Calcium acetate hydrate	0.1 M Tris pH 7.5	15 % w/v PEG 4000
2.10	0.2 M Calcium acetate hydrate		20 % w/v PEG 3350
2.30	0.2 M Calcium acetate hydrate	0.1 M Tris pH 7.5	8 % w/v PEG 20,000,<br>8 % v/v PEG 500 MME
2.60	0.2 M Calcium acetate hydrate	0.1 M Tris pH 7.5	25 % w/v PEG 2000 MME
**Crystal form 3*****			
3.0	8% v/v Tacsimate	pH 5.0	20 % w/v PEG 3350
3.6	0.2 M Ammonium chloride	pH 6.3	20 % w/v PEG 3350
~4	0.2 M Sodium malonate	pH 5.0	20 % w/v PEG 3350
Processing failed			
2.0	0.8 M Sodium formate	0.1 M Sodium acetate pH 5.5	25 % w/v PEG 2000 MME
~3	4% v/v Tacsimate		12 % w/v PEG 3350
~8	0.2 M Ammonium iodide	pH 6.2	20 % w/v PEG 3350
Untested crystals			
-	0.2 M Calcium acetate hydrate	0.1 M Sodium acetate pH 5.5	15 % w/v PEG 4000
-		0.1 M Sodium citrate pH 5.5	20 % w/v PEG 3000
-	0.2 M Lithium chloride	0.1 M Sodium acetate pH 5.0	20 % w/v PEG 6000
-	0.2 M Sodium phosphate	pH 4.7	20 % w/v PEG 3350
-	0.15 M Cesium chloride		20 % w/v PEG 3350
-	0.2 M Ammonium citrate	pH 5.1	20 % w/v PEG 3350
-	0.2 M Sodium sulfate	pH 6.7	20 % w/v PEG 3350
-	0.2 M Potassium sulfate	pH 6.8	20 % w/v PEG 3350
-	0.2 M Sodium bromide		20 % w/v PEG 3350
-	0.2 M Sodium iodide		20 % w/v PEG 3350
-	0.2 M Sodium thiocyanate		20 % w/v PEG 3350
-		0.1 M BIS-TRIS pH 6.5	25 % w/v PEG 3350
-		0.1 M BIS-TRIS pH 6.5	25 % w/v PEG 5000 MME
-	0.15 M Sodium thiocyanate	0.1 M Tris pH 8.5	20 % w/v PEG 1500
-	0.15 M Sodium thiocyanate	0.1 M Tris pH 8.5	20 % w/v PEG 600
-	0.15 M Sodium thiocyanate		20 % w/v PEG 500 MME
-	1.5 M Ammonium sulfate	0.1 M Tris pH 8.5	
-	1.5 M Ammonium sulfate	0.1 M Sodium acetate pH 5.5	
-	1.8 M Lithium sulfate	0.1 M Sodium acetate pH 5.5	

*Crystal form 1: C 2 2 2_1; a, b, c (Å); α, β, γ (°) = ~85, 88, 165; 90, 90, 90; 2 chains.
**Crystal form 2: $P4_3$ 2_1 2; a, b, c (Å); α, β, γ (°) = ~61, 61, 165; 90, 90, 90; 1 chain.
***Crystal form 3: $P4_1$ 2_1 2; a, b, c (Å); α, β, γ (°) = ~63, 63, 138; 90, 90, 90; 1 chain.

Table S2: FphH Q2G025 90% or higher sequence identical proteins listed in UniProt.

Organism/Strain	Gene	UniProt	NCBI	Ref	% Identity
S. aureus (strain NCTC 8325 / PS 47)	SAOUHSC_00802	Q2G025	YP_499358.1, WP_001165952.1	[1-3]	
S. aureus (strain COL)	*est* (SAUSA300_0763) (SACOL0845)	A0A0H2WWK7	WP_001165952.1	[4-6]	100
S. aureus (strain MSSA 1112 1)	*est*_1, *est*_2	A0A0D6HZA6	WP_001165952.1		100
S. aureus strain USA300 (NRS384)	*est* (SAUSA300_0763)	A0A0H2XJL0	WP_001165952.1	[4]	100
S. aureus (strain Newman)	NWMN_0748	A0A0H3K7L4	WP_001165952.1	[7-8]	100
S. aureus subsp. aureus PSP1996	SA1_36049	A0A7U9J6W6	WP_001165952.1		100
S. aureus (strain UA930)	G0V76_11280	A0A6B5M3L9	WP_001165964.1		>90
S. schleiferi	*est*_3	A0A7Z7QYE5			>90
S. aureus subsp. aureus 55/2053	SAAG_01204	A0A8D9W304			>90
S. aureus subsp. aureus WW2703/97	SAYG_00664	A0A8D9ZEE1			>90
S. aureus subsp. aureus A017934/97	SHAG_02151	A0A8D9ZRL7			>90
S. aureus subsp. aureus M809	SAZG_00762	A0A8E0BHE3			>90
S. aureus	*est*_1	A0A8G2M7Y0			>90
S. aureus subsp. aureus MN8	*est*	A0A0E1X7F4	WP_001165967.1		>90
S. aureus (strain UA879)	*yva*K	A0A0U1MG85	WP_001165966.1		>90
S. aureus (strain SH44)	*est*_2	A0A2S6D6Q7	WP_001165970.1		>90
S. argenteus (strain NCTC13711)	*yva*K	A0A7U7JTA8	WP_001165963.1		>90
S. aureus subsp. aureus WBG10049	SAXG_01753	A0A7U8SQS9	WP_001165967.1		>90
S. aureus subsp. aureus 68-397	SACG_01685	A0A8D9STW1			>90
S. aureus subsp. aureus M876	SAEG_01686	A0A8D9T2M5			>90
S. aureus subsp. aureus Btn1260	SDAG_02176	A0A8D9ZK93			>90

S. schweitzeri (strain FSA096)	*est*_2	A0A077ULD3	WP_047532244.1		>90
S. aureus	*est*_2	A0A380E1L7			>90
S. aureus (strain 4578)	GAY51_03845, GO782_16175	A0A6A9GTQ8	WP_001165974.1		>90
S. aureus (strain UA850)	G0Y40_09770, G0Y58_13435, SAJPND4_00788	A0A6B5EMX5	WP_001165956.1		>90
S. aureus	G0Y31_06555	A0A6G4N4Y8			>90
S. aureus subsp. aureus M013	M013TW_0770	A0A7U4ASR0	WP_001165974.1		>90
S. aureus subsp. aureus 65-1322	SABG_02120	A0A7U8F758	WP_001165967.1		>90
S. simiae CCM 7213 = CCUG 51256	SS7213T_01691	G5JFX4	WP_002461957.1		>90
S. aureus subsp. aureus 71193	ST398NM01_0858	A0A0E0VM72	WP_001165970.1		>90
S. aureus subsp. aureus USA300_TCH959	*est*	A0A0E1VJC7	WP_001165957.1		>90
S. aureus (strain N315)	SA0734 (BAB41967)	A0A0H3JL94	WP_001165959.1	[9-11]	>90
S. aureus (strain UA847)	*est*_2	A0A0Z1B2B6	WP_001165967.1		>90
S. aureus (strain SZ-M4)	G6Y24_04510, GO793_01970, GO941_00845, HK402_04135	A0A390QSM5	WP_001165959.1		>90
S. aureus (strain UA802)	G0W85_07975, G0X68_08315, G0Y26_03275, G0Y30_08155, G0Y33_07915, G0Z32_11425, G0Z62_02705, HUW54_04015	A0A6B5UTZ1	WP_001165962.1	[12-13]	>90
S. aureus (strain MRSA252)	*est*	A0A7U7EUM1	WP_001165967.1		>90
S. aureus subsp. aureus D139	SATG_01647	A0A8D9Z412			>90
S. aureus subsp. aureus EMRSA16	SIAG_01895	A0A8E0BKB2			>90
S. aureus subsp. aureus C101	SARG_00860	A0A8E0DBD6			>90

S. aureus subsp. aureus 58-424	SCAG_01195	A0A8E0EEW6			>90
S. aureus subsp. aureus H19	SAUG_00271	A0A8S7HAH6			>90
S. aureus (strain Mu50 / ATCC 700699)	SAV0779	A0A0H3JU38	WP_001165959.1		>90
S. aureus (strain MW2)	MW0741	A0A0H3K2E1	WP_001165958.1		>90
S. schweitzeri (strain DSM 28300)	*est*_2	A0A2K4ANR4	WP_047551325.1		>90
S. aureus (strain UA915)	D7S40_10720, GZ128_01035, GZ156_13390, SA0759_00666, SA950122_00663	A0A641A7X1	WP_001165958.1		>90
S. aureus subsp. aureus ST228	SAI7S6_1006130	A0A7U7EYE4	WP_001165959.1		>90
S. aureus subsp. aureus C160	SFAG_02192	A0A7U8XRM1	WP_001165967.1		>90
E. coli (missannotated?)	E4K51_27650	A0A7X1T2S6			>90
S. aureus (strain UA759)	E4U00_01450	A0A7Z8DE98	WP_001165957.1		>90
S. aureus subsp. aureus E1410	SADG_02142	A0A8D9W6J8			>90
S. aureus subsp. aureus C427	SASG_02129	A0A8D9YZY7			>90
S. aureus subsp. aureus M899	SAWG_01378	A0A8E0DK35			>90
S. aureus subsp. aureus M1015	SAVG_01672	A0A8E0EFY1			>90
S. aureus subsp. aureus CN1	SAKOR_00783	T1Y896	WP_001165956.1		>90

Table S3. Protein structures similar to FphH.

Top10 Dali hits (lali is the number of aligned residue pairs). One additional structure from *Lactobacillus plantarum* identified via other searches and aligned in UCSF Chimera using 193 pairs.

#	Name	Organism	FphH %ID	PDB ID	Z-score	rmsd	lali[a]	Ref
**1**	PF00326	Synthetic construct	57	4DIU	39.8	1.1	243	-
**2**	Est30	*Geobacillus stearothermophilus / Bacillus stearothermophilus*	57	1TQH / 1R1D	39.5	1.1	240	[14]
**3**	BL28	*Bacillus licheniformis*	54	6NKG	39.2	1.2	243	[15]
**4**	EstD	*Lacticaseibacillus rhamnosus HN001*	28	3DKR, 3DLT, 3DYI, 3DYV, 3E1G	33.1	1.6	232	-
**5**	MGL	*Bacillus sp. H-257*	29	4KE6, 4KE7, 4KE8, 4KE9, 4KEA, 4LHE, 3RLI, 3RM3	32.4	1.5	209	[16-18]
**6**	BOMGL	*Cytobacillus oceanisediminis*	26	7E04	31.7	1.6	216	-
**7**	MGL	*Geobacillus sp. 12AMOR1, Cytobacillus oceanisediminis 2691*	27	7E0N	31.6	1.8	217	-
**8**	MGL	*Geobacillus sp. 12AMOR1*	27	5XKS	31.5	1.6	213	-
**9**	LipS[d]	Unknown	25	4FBL	30.8	2	215	[19]
**10**	MAGL*	*Homo sapiens*	20	6AX1, 6BQ0	25	2.3	212	[20-21]
**11**	LP0796	*Lactobacillus plantarum WCFS1*	29	7EBO		0.9	193	[22]

*: Human monoglyceride lipase converts monoacylglycerides to free fatty acids and glycerol.

Table S4. *In vitro* characterized lipases/carboxylesterases similar to FphH.

Name	Organism	Uniprot	GXSXG	FphH % identity	Substrates (Preference)	Dali	Ref
FphH	*Staphylococcus aureus*	Q2G025	GLSLG		C2-C10 (C4)		This work
EstA	*Geobacillus thermoleovorans*	Q2V6P5	GLSLG	58	C2-C12 (C2)		[23]
EstZH1	*Geobacillus sp.* ZH1	I3NWL3	GLSLG	58	C2-C12 (C2)		[24]
CEGk	*Geobacillus kaustophilus* HTA426	Q5KVF6	GLSLG	58	C2-C16 (C2)		[25]
Est30	*Geobacillus stearothermophilus*	Q06174	GLSLG	58	C2-C12 (C6)	#2	[26-27]
EstD9	*Anoxybacillus geothermalis* D9	A0A160FBT3	GLSLG	57	C2-C16 (C2)		[28]
EstOF4	*Bacillus pseudofirmus* OF4	D3FY90	GLSLG	56	C2-C12 (C8)		[29]
PDF1	*Anoxybacillus sp.* PDF1	G9C5W9	GLSLG	56	C4-C18 (C4)[a]		[30]
BL28	*Bacillus licheniformis*	Q65EQ1	GLSLG	56	C2-C12 (C2)	#3	[31]
YvaK	*Bacillus subtilis*	O32232	GLSLG	54	C2[b]		[27]
EstB2	*Bacillus sp.* 01-855	Q6DV84	GLSLG	54	C2-C12		[32]
LmH	*Listeria monocytogenes*	Q8Y4I9	GLSLG	52	C2-C12 (C4)		[33]
EstC1	*Bacillus coagulans*	Q6GV11	GLSLG	51	C3-C18 (C4)		[11]
EstUT1	*Ureibacillus thermosphaericus*	A0A223PZA8	GVSLG	43	C2-C8 (C2)		[34]
LP_0796	*Lactobacillus plantarum* WCSF1	F9UM18	GLSLG	29	C2-C16 (C4)	#11	[35]
EstGtA2	*Geobacillus thermodenitrificans*	A4IP20	GLSMG	29	C4-C16 (C8)		[36]
JY144	*Bacillus stearothermophilus*	A0A087LCL0	GLSMG	28	[c]		[37]
MGL	*Bacillus sp. H-257*	P82597	GLSMG	27	~C4-C18 (C12)[d]	#5	[38]
LipS	Unknown	-	GLSMG	25	C4-C18 (C8)	#9	[19]
FphB	*Staphylococcus aureus*	Q2FV90	GDSAG	23	C4-C8 (C4)		[8]
FphF	*Staphylococcus aureus*	Q2FUY3	GHSMG	17	C2-C10 (C7)		[39]

a: Only C4, C12, C18 tested.

b: Only C2 tested.

c: Only (R,S)-ketoprofen ethyl ester tested.

d: Only various monoacylglycerols C4-C18 tested, highest activity for C12:0 1-Monolauroylglycerol.

Table S5. Model peptide substrates IQ1-IQ8 that did not have any cleavage activity by FphH.

IQ1	Mca-His-Gln-Lys-Leu-Val-Phe-Phe-Ala-Lys(DNP)-NH2
IQ2	Mca-Glu-Val-Lys-Met-Asp-Ala-Glu-Phe-Lys(DNP)-NH2
IQ3	Mca-Ser-Glu-Val-Asn-Leu-Asp-Ala-Glu-Phe-Arg-Lys(DNP)-Arg-Arg-NH2
IQ4	Mca-Arg-Pro-Lys-Pro-Tyr-Ala-Nva-Trp-Met-Lys(DNP)-NH2
IQ5	Mca-Gly-Lys-Pro-Ile-Leu-Phe-Phe-Arg-Leu-Lys(DNP)-DArg-NH2
IQ6	Mca-Arg-Pro-Pro-Gly-Phe-Ser-Ala-Phe-Lys(DNP)-OH
IQ7	Mca-Val-Asp-Gln-Met-Asp-Gly-Trp-Lys-(DNP)-NH2
IQ8	Abz-Gly-Ile-Val-Arg-Ala-Lys(DNP)-OH

Table S6. Tryptic digested compound 3 bound FphH mass spectrometry (MSMS) analysis.

Annotated Sequence	Modifications Serine/Threonine	# PSMs	Positions in FphH	Abundances	XCorr (by Search Engine)
[K].GYTSYAPQYEGHAAPPDEILK.[S]	1×compound 3 [S/T]	3	45-65	1.94E+08	3.74
[K].DALDGYDYLVEQGYDEIVVAGL SLGGDFALK.[L]	1×compound 3 [S23]	12	74-104	4.89E+08	6.33

Table S7: FphH structure data collection and processing.

Values for the outer shell are given in parentheses.

PDB ID	8FTP
Diffraction source	Australian synchrotron MX2
Wavelength (Å)	0.954
Detector	DECTRIS EIGER X 16M
Space group	$C2\ 2\ 2_1$
a, b, c (Å)	85.15 87.84 164.70
α, β, γ (°)	90, 90, 90
Resolution range (Å)	49.09 – 1.37 (1.39 – 1.37)
Total No. of reflections	1,722,894 (81,791)
No. of unique reflections	129,324 (6,219)
Completeness (%)	99.8 (97.3)
Redundancy	13.3 (13.2)
$\langle I/\sigma(I)\rangle$	18.9 (1.9)
$CC_{1/2}$	0.999 (0.641)
$R_{merge.}$	0.062 (1.484)
$R_{p.i.m.}$	0.026 (0.609)

Table S8: FphH structure solution and refinement.

Values for the outer shell are given in parentheses.

PDB ID	8FTP
Resolution range (Å)	40.85 – 1.37 (1.38 – 1.37)
Final R_{cryst}	0.152 (0.286)
Final R_{free}	0.165 (0.323)
Protein residues	486
Ligands	8 Ca
Water	541
R.m.s. deviations	
Bonds (Å)	0.009
Angles (°)	1.053
Average B factors (Å^2)	31.6
Ligands	29.1
Water	37.4
Ramachandran plot	
Most favored (%)	97.5
Outlier (%)	0

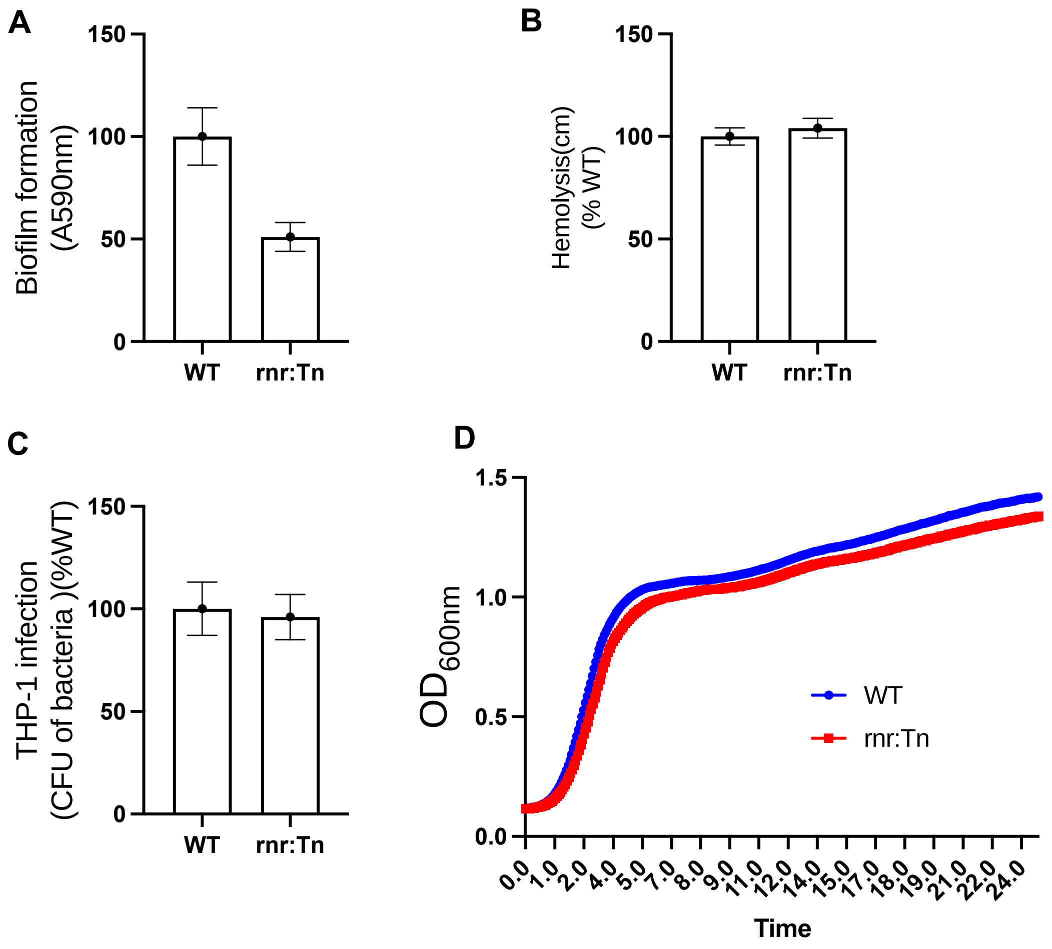

Figure S1: Functional characterization of *rnr*:Tn strain. A) Biofilm formation, B) Hemolysis of red blood cells, C) CFU of bacteria following THP-1 infection and D) growth curve of the JE2 WT and *rnr* transposon mutant (*rnr*:Tn). The values in A, B and C for the *rnr*:Tn were normalized to the WT at 100% with error bars indicating the SD of n=6 biological replicates. The growth curve of the WT and *rnr*:Tn shows the mean of n=8 independent experiments.

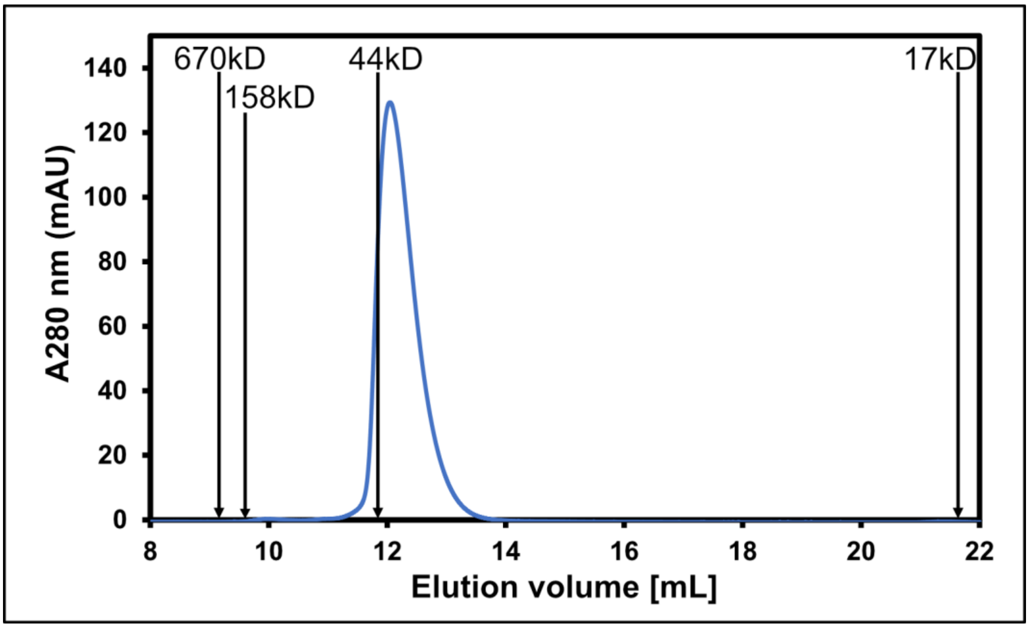

Figure S2: Gel-filtration chromatogram of FphH. A280 Superdex 75 chromatogram comparison of 28 kD FphH compared to molecular weight standard proteins Thyroglobulin 670 kD; γ-globulin 158 kD, Ovalbumin 44 kD and Myoglobin 17 kD.

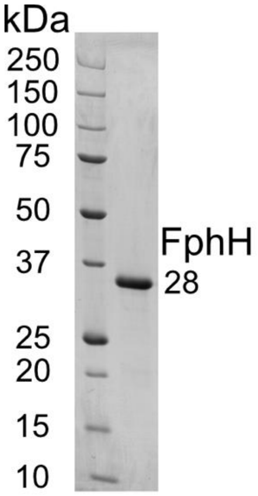

Figure S3: SDS-PAGE of FphH. Purified samples of FphH were loaded on a gradient 10-15% (w/v) polyacrylamide gel, resolved by SDS-PAGE, and detected by staining with Coomassie Brilliant Blue dye. Molecular weights of marker on the left compared to purified FphH, sample taken from gel-filtration illustrated in the previous figure.

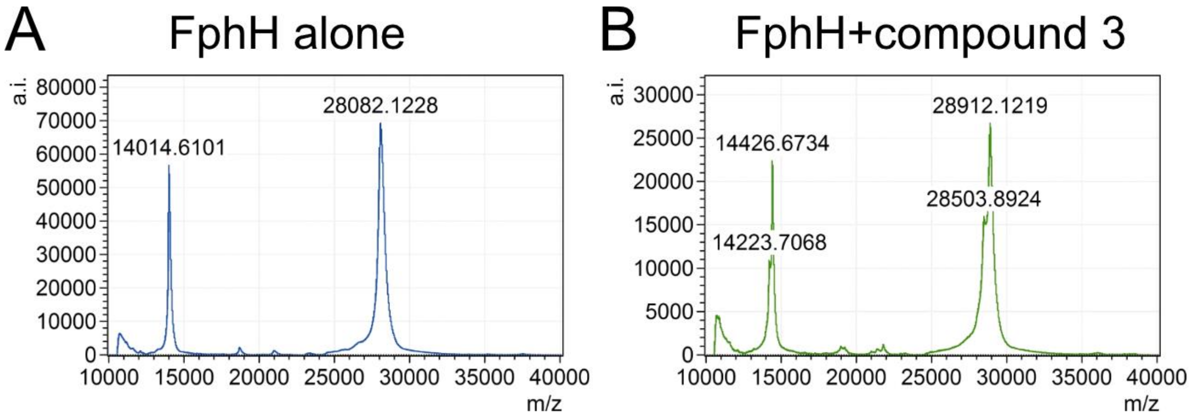

Figure S4: MALDI-TOF intact protein measurement. The spectra show the singly charged protein ions at about 28000 m/z and the doubly charged ions at about 14000 m/z. This low-resolution linear mode MALDI-TOF measurement has an estimated error of +/- 50 Da. A) Spectrum of purified full-length FphH, after cleavage of N-terminal His-tag form size exclusion peak shown in Fig. S1 and S2 (predicted molecular weight including three remaining N-terminal residues GPG from His-tag is 28305.68 Da). B) Spectrum of purified full-length FphH incubated with compound 3 (molecular weight of 417.13 Da) overnight indicates singly and doubly modified FphH.

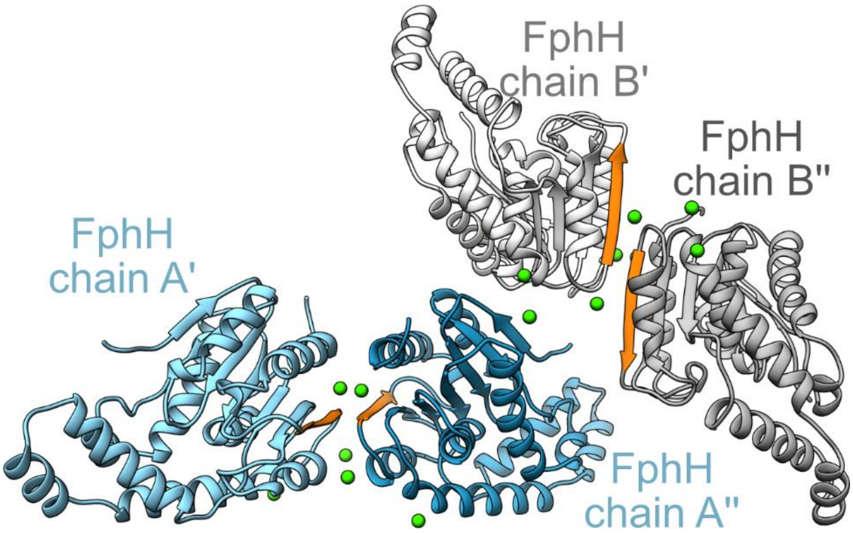

Figure S5: Calcium binding in the FphH crystal. FphH crystal structure (PDB ID 8FTP) shows Ca atoms (green) bound to the FphH surface facilitating crystal interfaces between symmetry copies of FphH chain A (blue/dark blue) and chain B (grey/dark grey). C-terminal β-strand at crystal interface in orange.

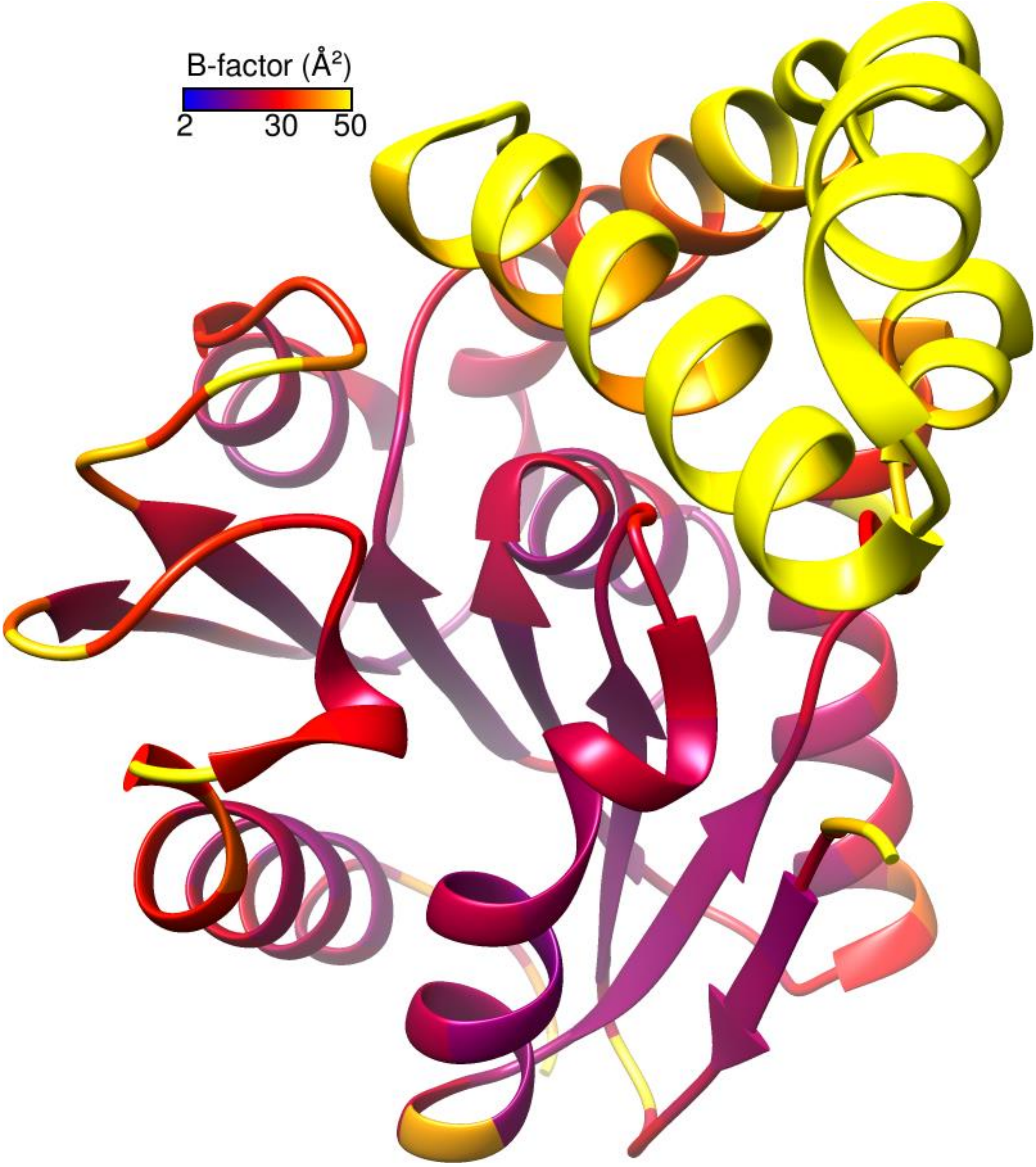

Figure S6: **FphH B-factor analysis.** Helices connecting β4 and β5 (residues 115-181) are shown in orange-yellow due to their higher B-factors compared with the rest of the structure.

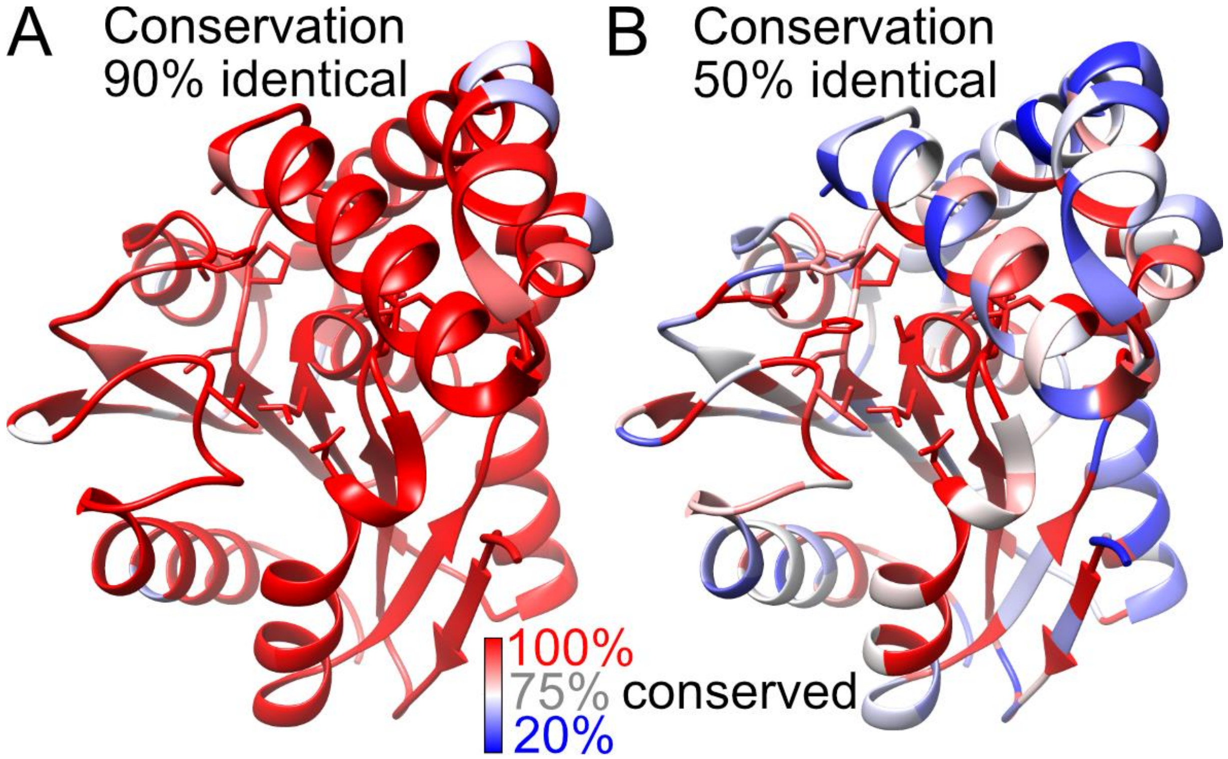

Figure S7: FphH structural conservation. Sequence alignment of FphH homologs mapped onto the FphH structure. Active site residues side chains are shown. Residues and ribbon colored according to shown key. A) 52 sequences at 90% identity to FphH (Q2G025). B) 1304 sequences at 50% identity to FphH (Q2G025).

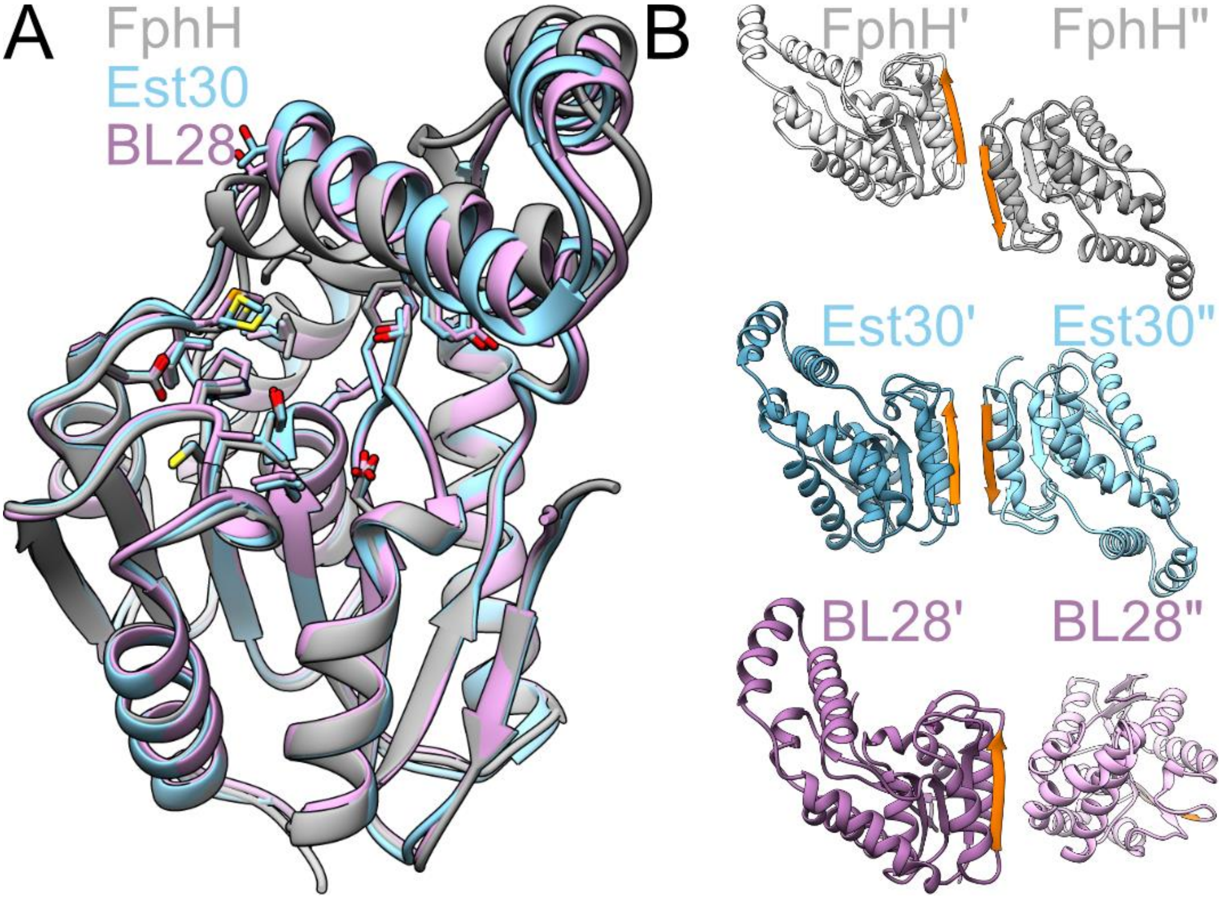

Figure S8: Comparison of FphH homologs. A) Alignment of grey FphH (PDB ID 8FTP), blue Est30 (1TQH) and pink BL28 (6NKG) with active side residue sidechains shown. B) Crystal symmetry copies of each homolog in the crystal structure with C-terminal β-strand in orange.

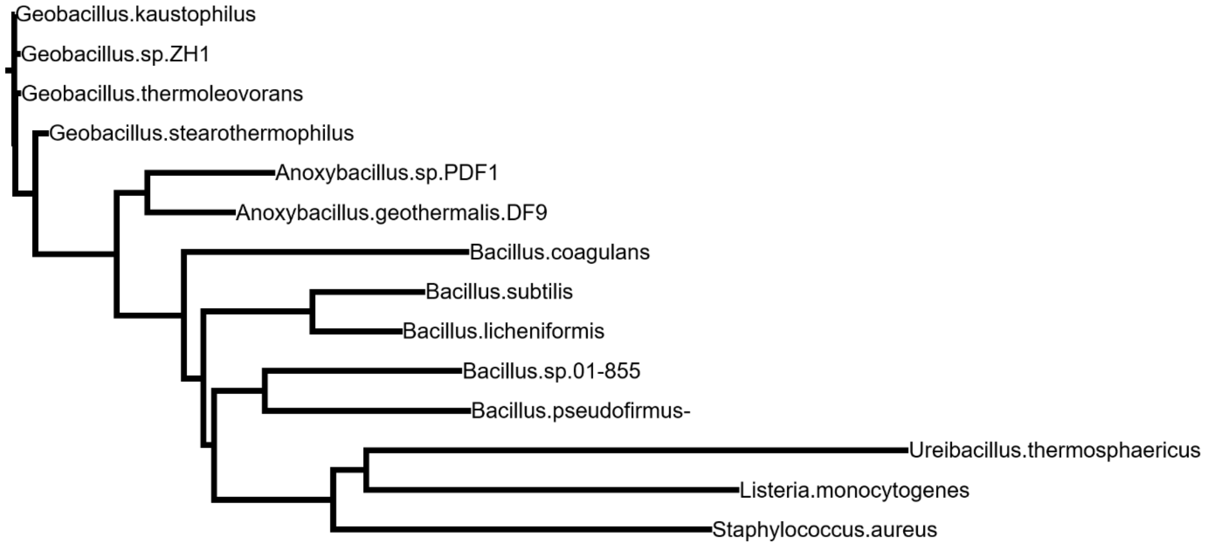

Figure S9: Phylogenetic tree of Table S4 homologous family of *S. aureus* FphH.

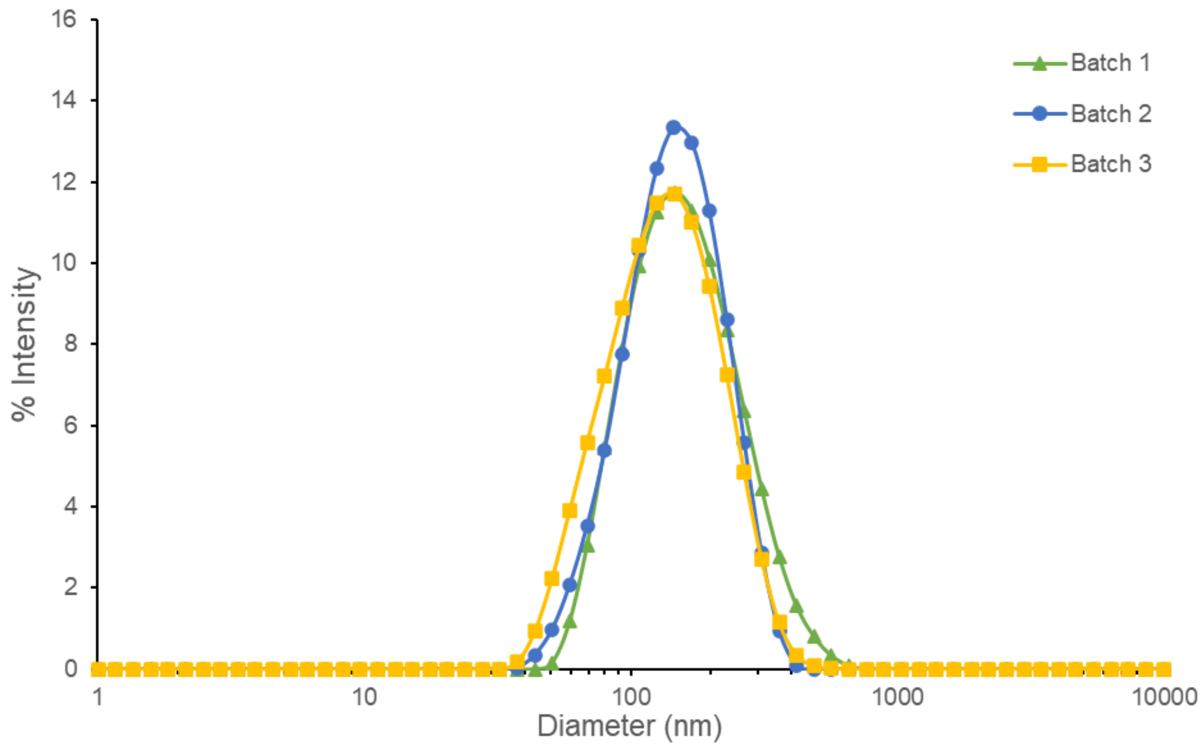

Figure S10: Dynamic light scattering (DLS) measurements of liposome batches. DLS analysis prior to incubation with FphH or PLA_2. Intensity–weighted size distributions of the three independently prepared CF loaded liposome batches. Samples were diluted to a lipid concentration of 0.25 mg/mL in pH 7.4 1X PBS. Polydispersity index (PDI) of the three samples were all < 0.2.

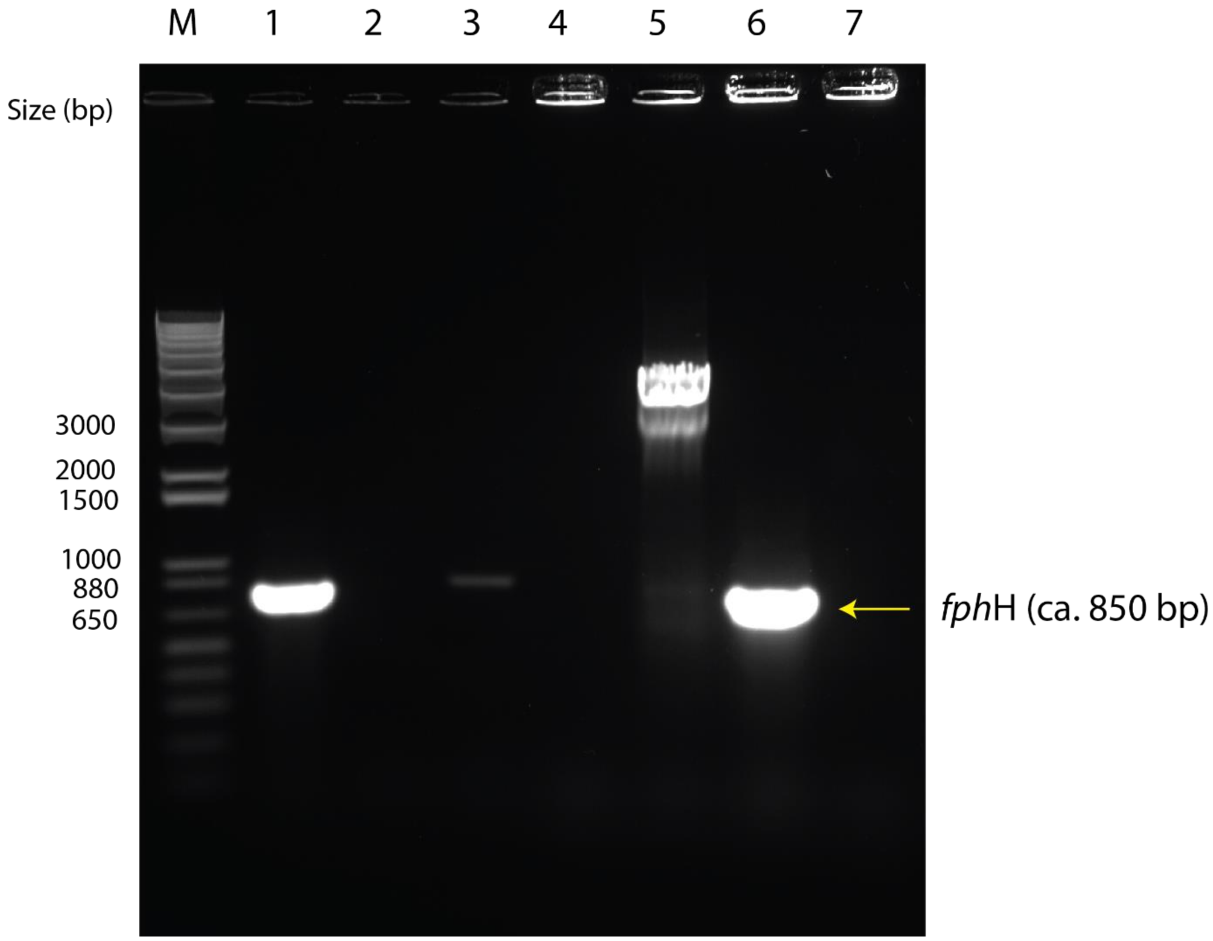

Figure S11: PCR-validation of *fph*H:Tn strains. PCR-products of an *fph*H gene-specific PCR (*fph*H_Fw: AAGTATGCAAAGGGAGCGTT, *fph*H_rv:TCATTATGCCCCTCCTTTCT reaction using were analyzed by agarose-gel electrophoresis after amplification with Phire Green Hot Start II DNA Polymerase. M: Marker, 1. Newman 2. Newman *fph*H:Tn, 3. Newman pTNT-*fph*H, 4. LAC_*fph*H:Tn, 5. ATCC35556_*fph*H:Tn, 6: SH1000, 7. SH1000_*fph*H:Tn. The arrow indicates the location of the amplified *fph*H band, which is absent in *fph*H-transposon mutant strains.

Supporting information references

1. Yang, D.; Jin, Y.; He, X.; Dong, A.; Wang, J.; Wu, R., Inferring multilayer interactome networks shaping phenotypic plasticity and evolution. *Nat Commun* **2021,** *12*, 5304. DOI: 10.1038/s41467-021-25086-5.

2. Fellner, M., Newly discovered Staphylococcus aureus serine hydrolase probe and drug targets. *ADMET DMPK* **2022,** *10*, 107-114. DOI: 10.5599/admet.1137.

3. Keller, L. J.; Lentz, C. S.; Chen, Y. E.; Metivier, R. J.; Weerapana, E.; Fischbach, M. A.; Bogyo, M., Characterization of Serine Hydrolases Across Clinical Isolates of Commensal Skin Bacteria Staphylococcus epidermidis Using Activity-Based Protein Profiling. *ACS Infect Dis* **2020,** *6*, 930-938. DOI: 10.1021/acsinfecdis.0c00095.

4. Reed, P.; Sorg, M.; Alwardt, D.; Serra, L.; Veiga, H.; Schäper, S.; Pinho, M. G., A CRISPRi-based genetic resource to study essential *Staphylococcus aureus* genes. *bioRxiv* **2022**, 2022.2010.2031.514627. DOI: 10.1101/2022.10.31.514627.

5. Delgado, A.; Zaman, S.; Muthaiyan, A.; Nagarajan, V.; Elasri, M. O.; Wilkinson, B. J.; Gustafson, J. E., The fusidic acid stimulon of Staphylococcus aureus. *J Antimicrob Chemother* **2008,** *62*, 1207-1214. DOI: 10.1093/jac/dkn363.

6. Miller, H. K. In *Characterization of the Lone Extracytoplasmic Function Sigma Factor, deltaS, and its Role in the Staphylococcus aureus Virulence and Stress Responses*, 2012.

7. Tsuchiya, Y.; Zhyvoloup, A.; Bakovic, J.; Thomas, N.; Yu, B. Y. K.; Das, S.; Orengo, C.; Newell, C.; Ward, J.; Saladino, G.; Comitani, F.; Gervasio, F. L.; Malanchuk, O. M.; Khoruzhenko, A. I.; Filonenko, V.; Peak-Chew, S. Y.; Skehel, M.; Gout, I., Protein CoAlation and antioxidant function of coenzyme A in prokaryotic cells. *Biochem J* **2018,** *475*, 1909-1937. DOI: 10.1042/BCJ20180043.

8. Lentz, C. S.; Sheldon, J. R.; Crawford, L. A.; Cooper, R.; Garland, M.; Amieva, M. R.; Weerapana, E.; Skaar, E. P.; Bogyo, M., Identification of a *S. aureus* virulence factor by activity-based protein profiling (ABPP). *Nat Chem Biol* **2018,** *14*, 609-617. DOI: 10.1038/s41589-018-0060-1.

9. Ben Zakour, N. L.; Sturdevant, D. E.; Even, S.; Guinane, C. M.; Barbey, C.; Alves, P. D.; Cochet, M. F.; Gautier, M.; Otto, M.; Fitzgerald, J. R.; Le Loir, Y., Genome-wide analysis of ruminant Staphylococcus aureus reveals diversification of the core genome. *J Bacteriol* **2008,** *190*, 6302-6317. DOI: 10.1128/JB.01984-07.

10. Said Salim, B. The role of Rot in the regulatory cascade of *Staphylococcus aureus* virulence genes. Ph.D., New York University, United States -- New York, 2002.

11. Mnisi, S. M.; Louw, M. E.; Theron, J., Cloning and characterization of a carboxylesterase from Bacillus coagulans 81-11. *Curr Microbiol* **2005,** *50*, 196-201. DOI: 10.1007/s00284-004-4423-3.

12. Yu, J. B.; Zhao, Z. X.; Peng, R.; Pan, L. B.; Fu, J.; Ma, S. R.; Han, P.; Cong, L.; Zhang, Z. W.; Sun, L. X.; Jiang, J. D.; Wang, Y., Gut Microbiota-Based Pharmacokinetics and the Antidepressant Mechanism of Paeoniflorin. *Front Pharmacol* **2019,** *10*, 268. DOI: 10.3389/fphar.2019.00268.

13. Zhao, Z. X.; Fu, J.; Ma, S. R.; Peng, R.; Yu, J. B.; Cong, L.; Pan, L. B.; Zhang, Z. G.; Tian, H.; Che, C. T.; Wang, Y.; Jiang, J. D., Gut-brain axis metabolic pathway regulates antidepressant efficacy of albiflorin. *Theranostics* **2018,** *8*, 5945-5959. DOI: 10.7150/thno.28068.

14. Liu, P.; Wang, Y. F.; Ewis, H. E.; Abdelal, A. T.; Lu, C. D.; Harrison, R. W.; Weber, I. T., Covalent reaction intermediate revealed in crystal structure of the Geobacillus stearothermophilus carboxylesterase Est30. *J Mol Biol* **2004,** *342*, 551-561. DOI: 10.1016/j.jmb.2004.06.069.

15. Ju, H.; Pandian, R.; Kim, K.; Kim, K. K.; Kim, T. D., Crystallization and preliminary X-ray analysis of a novel type of lipolytic hydrolase from Bacillus licheniformis. *Acta Crystallogr F Struct Biol Commun* **2014,** *70*, 473-475. DOI: 10.1107/S2053230X14004142.

16. Rengachari, S.; Bezerra, G. A.; Riegler-Berket, L.; Gruber, C. C.; Sturm, C.; Taschler, U.; Boeszoermenyi, A.; Dreveny, I.; Zimmermann, R.; Gruber, K.; Oberer, M., The structure of monoacylglycerol lipase from Bacillus sp H257 reveals unexpected conservation of the cap architecture between bacterial and human enzymes. *Bba-Mol Cell Biol L* **2012,** *1821*, 1012-1021. DOI: 10.1016/j.bbalip.2012.04.006.

17. Rengachari, S.; Aschauer, P.; Schittmayer, M.; Mayer, N.; Gruber, K.; Breinbauer, R.; Birner-Gruenberger, R.; Dreveny, I.; Oberer, M., Conformational Plasticity and Ligand Binding of Bacterial Monoacylglycerol Lipase. *Journal of Biological Chemistry* **2013,** *288*, 31093-31104. DOI: 10.1074/jbc.M113.491415.

18. Tsurumura, T.; Tsuge, H., Substrate selectivity of bacterial monoacylglycerol lipase based on crystal structure. *J Struct Funct Genomics* **2014,** *15*, 83-89. DOI: 10.1007/s10969-014-9181-2.

19. Chow, J.; Kovacic, F.; Dall Antonia, Y.; Krauss, U.; Fersini, F.; Schmeisser, C.; Lauinger, B.; Bongen, P.; Pietruszka, J.; Schmidt, M.; Menyes, I.; Bornscheuer, U. T.; Eckstein, M.; Thum, O.; Liese, A.; Mueller-Dieckmann, J.; Jaeger, K. E.; Streit, W. R., The metagenome-derived enzymes LipS and LipT increase the diversity of known lipases. *PLoS One* **2012,** *7*, e47665. DOI: 10.1371/journal.pone.0047665.

20. Butler, C. R.; Beck, E. M.; Harris, A.; Huang, Z.; McAllister, L. A.; Ende, C. W. A.; Fennell, K.; Foley, T. L.; Fonseca, K.; Hawrylik, S. J.; Johnson, D. S.; Knafels, J. D.; Mente, S.; Noell, G. S.; Pandit, J.; Phillips, T. B.; Piro, J. R.; Rogers, B. N.; Samad, T. A.; Wang, J. E.; Wan, S. Y.; Brodney, M. A., Azetidine and Piperidine Carbamates as Efficient, Covalent Inhibitors of Monoacylglycerol Lipase. *Journal of Medicinal Chemistry* **2017,** *60*, 9860-9873. DOI: 10.1021/acs.jmedchem.7b01531.

21. McAllister, L. A.; Butler, C. R.; Mente, S.; O'neil, S. V.; Fonseca, K. R.; Piro, J. R.; Cianfrogna, J. A.; Foley, T. L.; Gilbert, A. M.; Harris, A. R.; Helal, C. J.; Johnson, D. S.; Montgomery, J. I.; Nason, D. M.; Noell, S.; Pandit, J.; Rogers, B. N.; Samad, T. A.; Shaffer, C. L.; da Silva, R. G.; Uccello, D. P.; Webb, D.; Brodney, M. A., Discovery of Trifluoromethyl Glycol Carbamates as Potent and Selective Covalent Monoacylglycerol Lipase (MAGL) Inhibitors for Treatment of Neuroinflammation. *Journal of Medicinal Chemistry* **2018,** *61*, 3008-3026. DOI: 10.1021/acs.jmedchem.8b00070.

22. Zhang, H.; Wen, B.; Liu, Y.; Du, G.; Wei, X.; Imam, K.; Zhou, H.; Fan, S.; Wang, F.; Wang, Y.; Xin, F., A reverse catalytic triad Asp containing loop shaping a wide substrate binding pocket of a feruloyl esterase from Lactobacillus plantarum. *Int J Biol Macromol* **2021,** *184*, 92-100. DOI: 10.1016/j.ijbiomac.2021.06.033.

23. Soliman, N. A.; Knoll, M.; Abdel-Fattah, Y. R.; Schmid, R. D.; Lange, S., Molecular cloning and characterization of thermostable esterase and lipase from Geobacillus thermoleovorans YN isolated from desert soil in Egypt. *Process Biochem* **2007,** *42,* 1090-1100. DOI: 10.1016/j.procbio.2007.05.005.

24. Zhu, Y. B.; Liu, G. M.; Li, H. B.; Liu, J. W.; Bai, X. M.; Guan, R.; Cai, H. N., Cloning and characterization of a thermostable carboxylesterase from inshore hot spring thermophile Geobacillus sp ZH1. *Acta Oceanol Sin* **2012,** *31,* 117-126. DOI: 10.1007/s13131-012-0258-0.

25. Montoro-Garcia, S.; Martinez-Martinez, I.; Navarro-Fernandez, J.; Takami, H.; Garcia-Carmona, F.; Sanchez-Ferrer, A., Characterization of a Novel Thermostable Carboxylesterase from Geobacillus kaustophilus HTA426 Shows the Existence of a New Carboxylesterase Family. *Journal of Bacteriology* **2009,** *191,* 3076-3085. DOI: 10.1128/Jb.01060-08.

26. Ewis, H. E.; Abdelal, A. T.; Lu, C. D., Molecular cloning and characterization of two thermostable carboxyl esterases from Geobacillus stearothermophilus. *Gene* **2004,** *329,* 187-195. DOI: 10.1016/j.gene.2003.12.029.

27. Henke, E.; Bornscheuer, U. T., Esterases from Bacillus subtilis and B-stearothermophilus share high sequence homology but differ substantially in their properties. *Appl Microbiol Biot* **2002,** *60,* 320-326. DOI: 10.1007/s00253-002-1126-1.

28. Johan, U. U. M.; Rahman, R.; Kamarudin, N. H. A.; Latip, W.; Ali, M. S. M., A new hyper-thermostable carboxylesterase from Anoxybacillus geothermalis D9. *Int J Biol Macromol* **2022,** *222,* 2486-2497. DOI: 10.1016/j.ijbiomac.2022.10.033.

29. Rao, L.; Xue, Y.; Zheng, Y.; Lu, J. R.; Ma, Y., A novel alkaliphilic bacillus esterase belongs to the 13(th) bacterial lipolytic enzyme family. *PLoS One* **2013,** *8,* e60645. DOI: 10.1371/journal.pone.0060645.

30. Ay, F.; Karaoglu, H.; Inan, K.; Canakci, S.; Belduz, A. O., Cloning, purification and characterization of a thermostable carboxylesterase from Anoxybacillus sp. PDF1. *Protein Expr Purif* **2011,** *80,* 74-79. DOI: 10.1016/j.pep.2011.06.019.

31. Ju, H.; Jang, E.; Ryu, B. H.; Kim, T. D., Characterization and preparation of highly stable aggregates of a novel type of hydrolase (BL28) from Bacillus licheniformis. *Bioresour Technol* **2013,** *128*, 81-86. DOI: 10.1016/j.biortech.2012.10.016.

32. Karpushova, A.; Brummer, F.; Barth, S.; Lange, S.; Schmid, R. D., Cloning, recombinant expression and biochemical characterisation of novel esterases from Bacillus sp. associated with the marine sponge Aplysina aerophoba. *Appl Microbiol Biotechnol* **2005,** *67*, 59-69. DOI: 10.1007/s00253-004-1780-6.

33. Ju, H.; Ryu, B. H.; Doohun Kim, T., Identification, characterization, immobilization of a novel type hydrolase (LmH) from Listeria monocytogenes. *Int J Biol Macromol* **2015,** *72*, 63-70. DOI: 10.1016/j.ijbiomac.2014.07.058.

34. Sorokina, K. N.; Samoylova, Y. V.; Parmon, V. N., Thermostable Esterase estUT1 from Ureibacillus thermosphaericus: Effect of TrxA Tag on the Enzyme Properties. *Catalysis in Industry* **2020,** *12*, 148-154. DOI: 10.1134/S2070050420020099.

35. Esteban-Torres, M.; Reveron, I.; Mancheno, J. M.; de Las Rivas, B.; Munoz, R., Characterization of a feruloyl esterase from Lactobacillus plantarum. *Appl Environ Microbiol* **2013,** *79*, 5130-5136. DOI: 10.1128/AEM.01523-13.

36. Charbonneau, D. M.; Meddeb-Mouelhi, F.; Beauregard, M., A novel thermostable carboxylesterase from Geobacillus thermodenitrificans: evidence for a new carboxylesterase family. *J Biochem* **2010,** *148*, 299-308. DOI: 10.1093/jb/mvq064.

37. Kim, J. Y.; Choi, G. S.; Kim, Y. J.; Ryu, Y. W.; Kim, G. J., A new isolate Bacillus stearothermophilus JY144 expressing a novel esterase with high enantioselectivity to (R)-ketoprofen ethyl ester: strain selection and gene cloning. *J Mol Catal B-Enzym* **2002,** *18*, 133-145. DOI: 10.1016/S1381-1177(02)00078-4.

38. Kitaura, S.; Suzuki, K.; Imamura, S., Monoacylglycerol lipase from moderately thermophilic Bacillus sp. strain H-257: molecular cloning, sequencing, and expression in Escherichia coli of the gene. *J Biochem* **2001,** *129*, 397-402. DOI: 10.1093/oxfordjournals.jbchem.a002870.

39. Fellner, M.; Lentz, C. S.; Jamieson, S. A.; Brewster, J. L.; Chen, L.; Bogyo, M.; Mace, P. D., Structural Basis for the Inhibitor and Substrate Specificity of the Unique Fph Serine Hydrolases of Staphylococcus aureus. *ACS Infect Dis* **2020,** *6*, 2771-2782. DOI: 10.1021/acsinfecdis.0c00503.
